# *Paramixta manurensis* gen. nov., sp. nov., a novel member of the family *Erwiniaceae* producing indole-3-acetic acid isolated from mushroom compost

**DOI:** 10.1038/s41598-024-65803-w

**Published:** 2024-07-05

**Authors:** Jueun Kim, Hyosuk Yun, Aminallah Tahmasebi, Jiyoung Nam, Ha Pham, Yong-Hak Kim, Hye Jung Min, Chul Won Lee

**Affiliations:** 1 Department of Chemistry, Chonnam National University, Gwangju, 61186 Republic of Korea; 2 Research Center, DAESANG InnoPark, Gangseo-gu, Seoul, 07789 Republic of Korea; 3 Department of Agriculture, Minab Higher Education Center, University of Hormozgan, Bandar Abbas, Iran; 4 Institute of Well-Aging Medicare & CSU G-LAMP Project Group, Chosun University, Gwangju, 61452 Republic of Korea; 5 Department of Microbiology, Daegu Catholic University School of Medicine, Daegu, 42472 Republic of Korea; 6 Department of Cosmetic Science, Gwangju Women’s University, Gwangju, 62396 Republic of Korea

**Keywords:** Chemical biology, Microbiology

## Abstract

There are numerous species in the *Erwiniaceae* family that are important for agricultural and clinical purposes. Here we described the *Erwiniaceae* bacterium PD-1 isolated from mushroom (*Pleurotus eryngii*) compost. Comparative genomic and phylogenetic analyses showed that the strain PD-1 was assigned to a new genus and species, *Paramixta manurensis* gen. nov., sp. nov. in the family *Erwiniaceae*. From the average amino acid index, we identified the five AroBEKAC proteins in the shikimate pathway as a minimal set of molecular markers to reconstruct the phylogenetic tree of the *Erwiniaceae* species. The strain PD-1 containing annotated genes for ubiquinone and menaquinone produced a higher level of ubiquinone (Q8) than demethylmenaquinone (DMK8) and menaquinone (MK8) in anaerobic condition compared to aerobic condition, as similarly did the reference strains from the genera *Mixta* and *Erwinia*. Results from fatty acid methyl ester and numerical analyses of strain PD-1 showed a similarity to species of the genera *Mixta* and *Winslowiella*. This study revealed that the strain’s ability to utilize polyols, such as glycerol, erythritol, and d-arabitol, distinguished the strain PD-1 from the nearest relative and other type strains. The analyzed genetic markers and biochemical properties of the strain PD-1 suggest its potential role in the process of mushroom compost through the degradation of carbohydrates and polysaccharides derived from fungi and plants. Additionally, it can produce a high concentration of indole-3-acetic acid as a plant growth-promoting agent.

## Introduction

The *Erwiniaceae* is a new family separated from the family *Enterobacteriaceae*^1^. The diverse members of *Erwiniaceae* belonging to the order *Enterobacterales* are Gram-negative, rod-shaped and non-spore forming microorganisms^1,2^. Since *Erwinia amylovora* was firstly proposed as a distinct taxon^3^, nine genera in the family *Erwiniaceae* have reportedly been given correct names: *Erwinia*^4^, *Tatumella*^5^, *Pantoea*^6^, *Buchnera*^7^, *Wigglesworthia*^8^, *Phaseolibacter*^9^, *Mixta*^10^, *Winslowiella*^11^, and *Duffyella*^12^. The taxonomic revision based on phylogenomic data changed the genus name from *Kalamiella piersonii* to *Pantoea piersonii*^12^. Additionally, *Izhakiella* and *Rosenbergiella* that had been classified as *Enterobacteriaceae* genera were reclassified to the genera of *Erwiniaceae*^13,14^. Members of *Erwiniaceae* have formed symbiotic or pathogenic relationships with plants, insects, humans, and animals in their natural environments and the International Space Station. There were a total of 1658 genome assemblies of *Erwiniaceae* at the National Center for Biotechnology Information (NCBI updated 24 Jan. 2024), which were so far clustered into 11 known genera, but 11 genomes could not validate the genus and species names. The genome sizes of most species range from 2.75 to 5.88 Mb, with a GC content of 44.4% to 58%. Genetic variations in endosymbiotic strains, such as *B. aphidicola* (0.64 Mb with 25.2% GC content), *W. glossinidia* (0.71 Mb with 23.8% GC content), *Candidatus Pantoea carbekii* (1.15 Mb with 30.6% GC content), *Candidatus Erwinia haradaeae* (1.09 Mb with 30.6% GC content) and *E. dacicola* (2.70 Mb with 52.8%), suggests a diversity of *Erwiniaceae* species.

Members of the *Erwiniaceae* are of agricultural and clinical importance. Despite the pathogenic variants, researchers explored a wide range of symbiotic relationship with the biological control of pests and diseases to benefit from sustainably managing agriculture and human health. *P. agglomerans* that causes plant diseases and also as opportunistic infections in humans has been considered as the most promising biocontrol agent for a variety of bacterial and fungal plant diseases^15^. *P. agglomerans* is able to produce the low-molecular-mass lipopolysaccharide (IP-PA1) that is effective to treat a variety of human and animal disorders, with a strong analgesic effect^16^. There are a rapidly increasing number of *Pantoea* isolates with diverse genes for industrial and agricultural applications in various hosts and environments, including soil and water^17^. Some *Pantoea* isolates are capable of producing antibiotics, such as pantocins, herbicolins, microcins, and phenazines, some of which are effective against the fire blight pathogen, *E. amylovora*^18,19^. Recent genomic approaches have provided new insights into the genetic diversity and the phylogenetic analysis of *Erwiniaceae*, particularly with a large number of uncharacterized or unknown genes in members of the genera of *Erwinia, Mixta, Pantoea,* and *Tatumella*^10,20–22^. A number of genome-based studies have been conducted to investigate the evolution of *Erwiniaceae* with different ecological, pathophysiological, and genetic features. However, a robust phylogeny including all genera of *Erwiniaceae* remains unresolved due to the high evolution rates of molecular markers and their variation across phylogenetic trees.

The object of this study was to characterize and classify the *Erwiniaceae* bacterium PD-1 isolated from mushroom compost. Genomic and phylogenetic analyses were conducted to reveal that strain PD-1 is the type strain of a new genus and species, named *Paramixta manurensis*. In this study, we identified AroBEKAC proteins in the shikimate pathway as a new molecular marker that links to the biosynthesis of respiratory quinone and cartenoid as chemotaxonomic markers. Further analyses showed that strain PD-1 has a potential role to play in the process of mushroom compost and production of indole-3-acetic acid (auxin), a characteristic hormone of plant growth-promoting bacteria.

## Results and discussion

### Isolation and identification of *Ewiniaceae* strain PD-1

**Figure 1:**
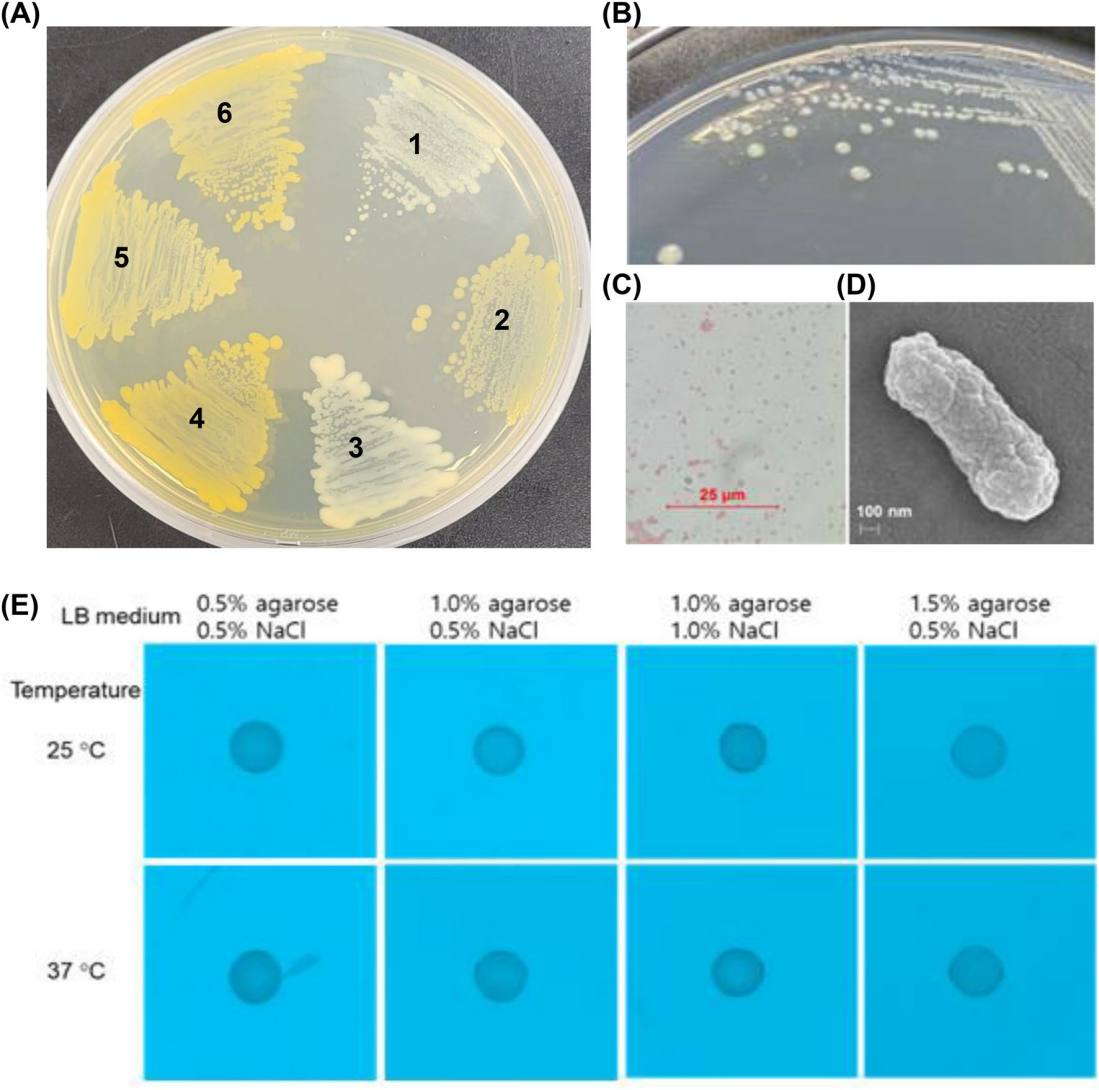
Morphology and motility of strain PD-1. (**A**) Comparison of colony color of strains: 1, strain PD-1; 2, *M. tenebrionis* KCTC 72449^T^; 3, *E. rhapontici* KACC 22740^T^; 4, *P. agglomerans* KACC 15275^T^; 5, *P. ananatis* KACC 22739^T^; and 6, *P. stewartii* KACC 22737^T^. (**B**) Morphology of colonies on a R2A plate. (**C**) Gram-negative cells at 1000× magnification under a light microscope. (**D**) Scanning electron microscopy (SEM) of gold–coated cells. (**E**) No motility of cells measured at 25 °C and 37 °C after spotting of a culture aliquot (5 μL) on LB media with different concentrations of Bacto agarose (0.5, 1.0, and 1.5%, w/v) and NaCl (0.5 and 1.0%, w/v).

Strain PD-1 was isolated from a mushroom culture compost, in order to obtain a native microorganism to develop a microbial agent for drilled grain waste composting and investigate its potential as a plant growth-promoting bacterium in mushroom compost. The isolate PD-1 formed a circular, convex, yellowish, non-sticky colony with 1.5–2.5 mm diameter on R2A agar plates, but the colony color was a pale yellow compared to *Pantoea* species’ color (Fig. 1). The Gram staining showed gram-negative rod, with a thickness of 0.3–0.5 µm and a length 0.9–1.2 µm measured by scanning electron microscopy. Optimal growth conditions of strain PD-1 were determined based on assessments of colony size and growth rate of cells, cultivated with aeration in R2A media at various temperature conditions from 10 to 40 °C (optimal at 30 °C), pH 5.0 to 10.0 (optimal at pH 7.0), and NaCl concentrations up to 6.5% (w/v, optimal at 0% NaCl). The cells neither showed motility in different LB medium culture conditions nor formed biofilms in the optimal conditions of the R2A medium.

A partial 16S rRNA gene sequence (GenBank accession number MN197605), obtained by PCR using genomic DNA of the strain PD-1 as template, showed the highest similarity (98.13%) to that of *P. vagans* LMG 241999^T^ (EF688012). A UPGMA phylogenetic tree indicates that strain PD-1 formed a separate branch between clades of *E. typographi*, *P. flectans* and *Rosenbergiella* species cluster (Supplementary Fig. S1). However, the large genera *Erwinia*, *Pantoea* and *Mixta* could not cluster with type strains, and endosymbiotic *B. aphidicola* and *W. glossinidia* comprised independent clades distant from the other outgroup *Brenneri* species belonging to the family *Pectobacteriaceae*. Thus, to improve the phylogeny of the *Erwiniaceae*, concatenated sequences of four house-keeping genes including *atpD*, *gyrB*, *infB* and *rpoB* genes were used for the multilocus sequence analysis (MLSA)^10–13, 23^. The MLSA phylogenetic tree shows that strain PD-1 was differentiated from the *Erwinia*-*Winslowiella* lineage, which was clearly distinguished from the *Mixta-Pantoea-Duffyella* lineage (Supplementary Fig. S2). This figure shows a close relation between the strain PD-1 and [*Pantoea*] *beijingensis* strain JZB2120001 (= LMG27579^T^), isolated from mushroom compost and the fruiting body of *P. eryngii*^24^. Previous studies suggested that *P. beijingensis* should be named as *E. beijingensis*^11,25^. The MLSA resulted in a high similarity of concatenated sequences with type strains to assign species more accurately than 16S, but it was still difficult to differentiate the genus *Winslowiella* from the genus *Erwinia*. The phylogenetic trees constructed based on the 16S and MLSA data were difficult to distinguish *Erwiniaceae* strains at the species and genus levels, so genomic and phenotypic approaches were mandatory for some strains.

### Genome analysis and discovery of new molecular marker

**Figure 2:**
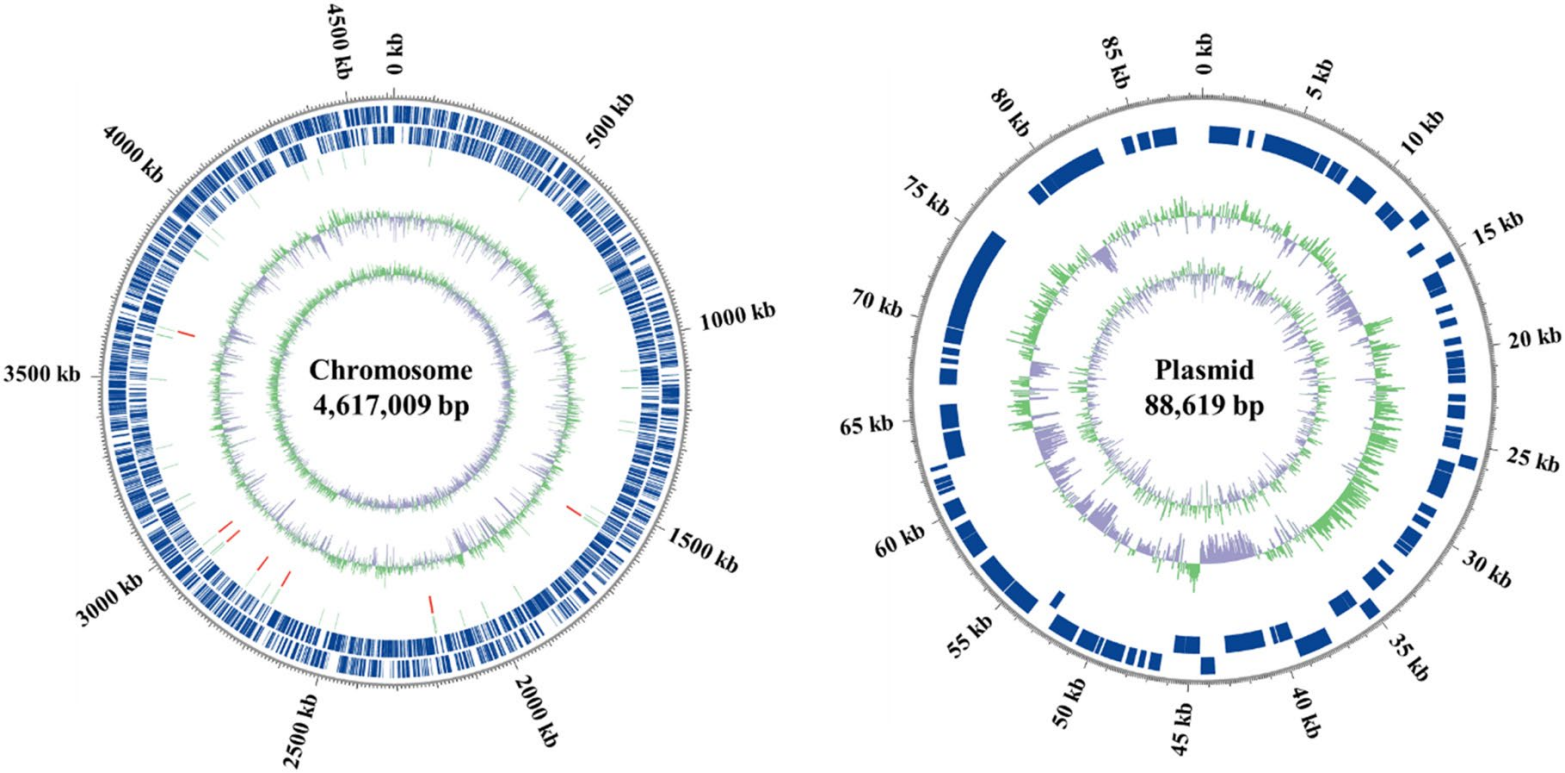
Circular genomic maps of chromosome and plasmid in strain PD-1. From outside to inside: position of circular DNA, forward and reverse coding sequences (CDSs), non-coding tRNA and rRNA at green and red bars, high and low GC levels in exterior green and interior lavender peaks from the average GC percentage of DNA, and GC skew, (G–C)/(G + C), showing higher G levels in exterior green peaks and higher C levels in interior lavender peaks.

Whole genome sequencing that was carried out on PacBio RSII and Illumina sequencing platforms led to the assembly of 2 contigs from 4,617,009 bp of a circular chromosome and 88,619 bp of a circular plasmid (Fig. 2). The hierarchical genome assembly process (HGAP) assembly was polished and corrected by aligning paired-end short reads from the PacBio and Illumina data. The NCBI Prokaryotic Genome Annotation Pipeline (PGAP) identified that the genome assembly of the strain PD-1 (accession number ASM1328538v1) contains 4525 genes including 4358 protein-coding sequences.

DNA-DNA hybridization (DDH) and average nucleotide index (ANI) values between whole genomes of strain PD-1 and type strains of *Erwiniaceae* were all lower than the standard cutoff values – 95% ANI and 70% DDH for species confirmation (Supplementary Data and Fig. S3). In the strain PD-1, approximately 20% of the genome sequence differed from that of the nearest relative [*Pantoea*] *beijingensis* JZB2120001 (Table 1, lower matrix). The pairwise genome comparisons of most species belonging to the genera *Erwinia* and *Pantoea* resulted in ANI > 96% and DDH > 70%^26^. The ANI values of all orthologous genes shared between strains of the same or closely related species have been estimated to range from 80 to 100%^27^. The high ANI values were used to verify species in prokaryote taxonomy by pairwise comparison with confirmed type strains of species with a default cutoff of 96% identity and 90% coverage^28^. The low genome sequence identity and coverage across endosymbiotic *B. aphidicola* and *W. glossinidia* assigned an invalid (zero) ANI value. Taken together, the ANI data indicated that strain PD-1 was a new species, but the genus cannot be defined by any pairwise genome comparison.

**Table 1:** A matrix of average nucleotide index (ANI) and average amino acid index (AAI) values between strain PD-1 and type strains of closely related species.

No	Strain names	1	2	3	4	5	6	7	8	9	10	11	12	13	14	15
1	Strain PD-1	100	81	79	79	79	79	79	79	79	78	76	77	76	77	77
2	[*Pantoea*] *beijingensis* JZB2120001	79	**100**	**82**	**81**	**81**	78	78	79	78	77	77	79	76	78	78
3	*Winslowiella iniecta* B120^T^	79	**80**	**100**	**86**	**86**	79	79	79	79	78	77	79	76	79	78
4	*Winslowiella arboricola* BAC 15a-03b^T^	79	**80**	**83**	**100**	**95**	78	79	79	79	78	77	79	76	78	78
5	*Winslowiella toletana* DAPP-PG 735^T^	79	**80**	**83**	**91**	**100**	78	79	79	78	77	77	79	76	78	78
6	*Mixta theicola* QC88-366^T^	79	79	79	79	80	**100**	**93**	**89**	**88**	**86**	76	77	75	76	76
7	*Mixta alhagi* LTYR-11Z^T^	79	79	79	79	79	**89**	**100**	**89**	**88**	**86**	76	77	75	77	76
8	*Mixta calida* DSM 22759^T^	79	79	79	79	80	**84**	**84**	**100**	**94**	**88**	76	77	75	77	76
9	*Mixta gaviniae* DSM 22758^T^	80	79	79	80	80	**84**	**83**	**90**	**100**	**87**	76	77	75	77	76
10	*Mixta tenebrionis* BIT-26^T^	79	79	79	79	79	**83**	**83**	**85**	**85**	**100**	75	75	74	76	76
11	*Erwinia oleae* DAPP-PG 531^T^	78	79	79	79	81	79	79	79	80	79	**100**	**80**	78	78	78
12	*Erwinia billingiae* Eb661^T^	79	79	80	80	80	79	79	79	79	79	**80**	**100**	80	80	79
13	*Erwinia psidii* IBSBF 435^T^	79	79	79	79	79	78	79	79	78	78	79	80	100	77	78
14	*Erwinia aphidicola* X001^T^	79	79	80	80	80	79	79	80	80	80	80	81	79	**100**	**86**
15	*Erwinia persicina* NBRC 102418^T^	78	79	80	79	79	79	79	79	79	79	79	80	79	**83**	**100**

The ANI-AAI matrix was reconstructed using ANI (lower matrix) and AAI (upper matrix) values between strain PD-1 and type strains of the family *Erwiniaceae* which were shown in the Supplementary Figs. S1 and S2. The species of the genera *Erwinia*, *Mixta* and *Winslowiella* were clustered according to the threshold values above 80% in the bold value boxes.

In order to assess the taxonomic position of the strain PD-1, average amino acid index (AAI) was determined between protein databases of strain PD-1 and type strains of *Erwiniaceae* (Supplementary Fig. S4). The AAI-based neighbor-joining tree has the cutoff value of 68% for genus delineation and the resulting genera are monophyletic. Based on this tree, the strain PD-1 was identified as a novel species of a new genus between the genera *Winslowella* and *Mixta*, for which the name *Paramixta manurensis* gen. nov., sp. nov. is proposed. Moreover, our analysis showed that the nearest relative [*Pantoea*] *beijingensis* JZB2120001 could better be named as species of the genus *Winslowella*^11^. Further studies may be needed for the classification of species belonging to the genera *Erwinia* and *Winslowella*.

**Figure 3:**
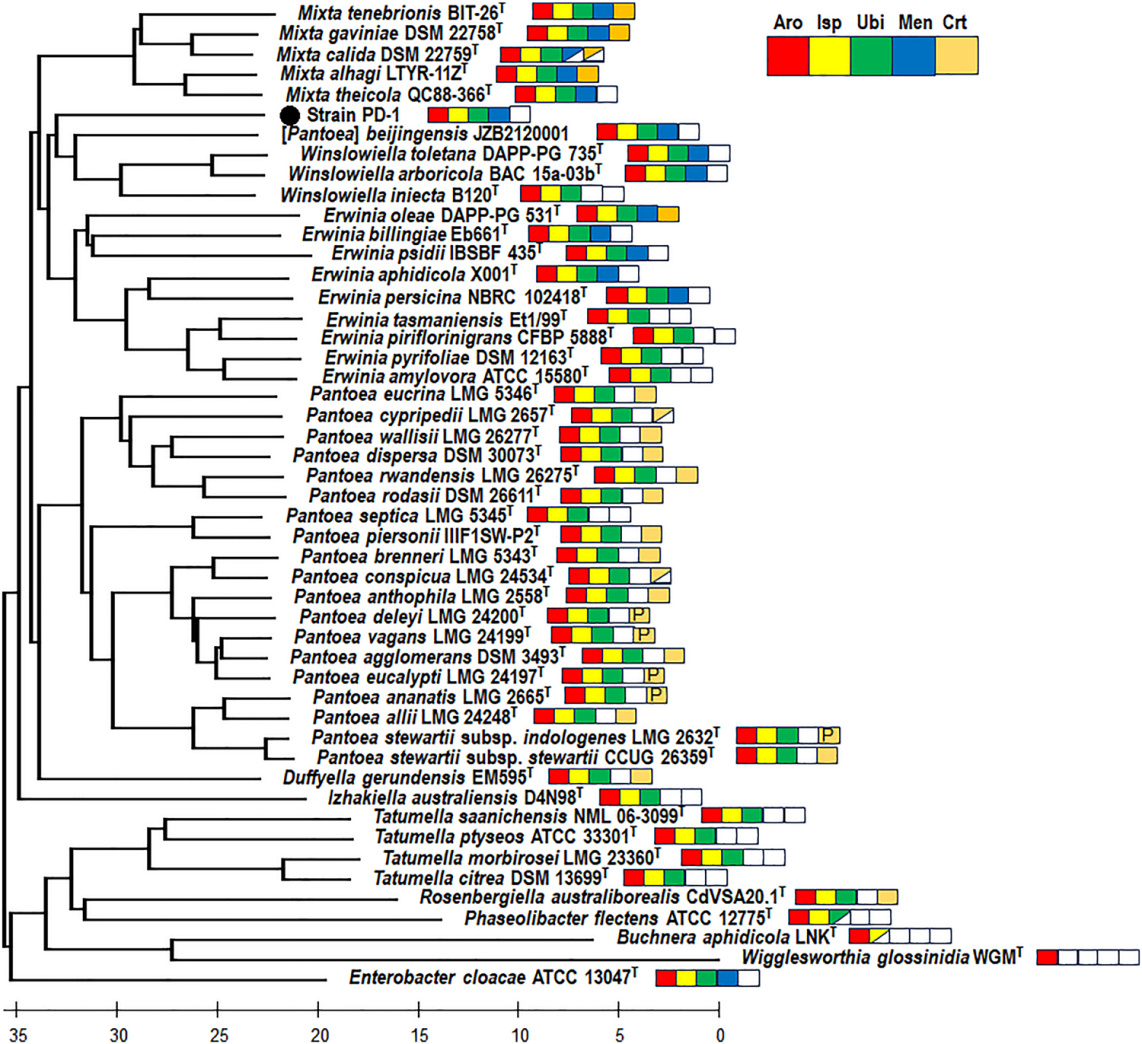
Average amino acid index-based neighbor-joining tree of strain PD-1 and type strains of *Erwiniaceae*. The tree was constructed based on AAI values, as shown in Supplementary Figure S4, and shows the genetic variants of chorismite (Aro), octaprenyl diphosphate (Isp), ubiquinone (Ubi), menaquinone (Men), and cartenoid (Crt) biosynthesis genes with colored boxes at the branch end of each strain. Half filled boxes and white boxes represent partial and total deficiencies in genome, respectively. Alphabet P included in yellow boxes of Crt indicates plasmid-encoded *crt* operons.

**Figure 4:**
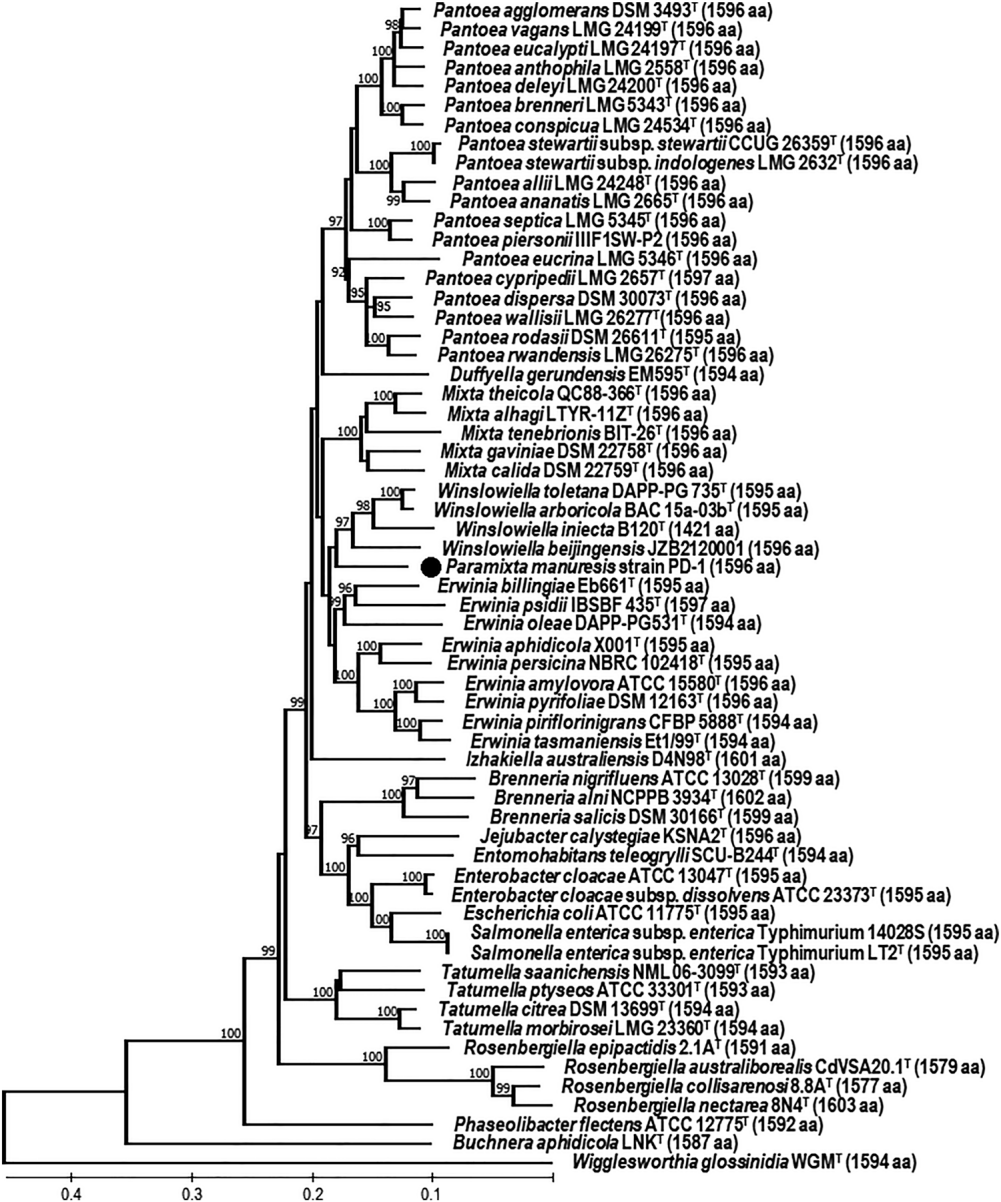
Minimum Evolution phylogenetic tree constructed based on concatenated sequences of AroBEKAC proteins in strain PD-1. The optimal tree was shown with more than 90% of 1000 replicate trees in the bootstrap test and drawn to scale, as the units of the number of amino acid substitutions per site, computed using the JTT matrix-based method and the Close-Neighbor-Interchange algorithm at a search level of 1 in MEGA X. There were a total of 1587 positions in the final dataset and the numbers of amino acid residues (aa) in concatenated AroBEKAC sequences are shown in parentheses.

In order to reconstruct the phylogeny of *Erwiniaceae*, five AroBEKAC proteins in the shikimate pathway were identified as a minimal set of molecular markers by gene ontology (GO) and Kyoto encyclopedia of genes and genomes (KEGG) analyses of the AAI data (Supplementary Data). The shikimate pathway is essential to lead to the synthesis of essential aromatic amino acids (e.g. tryptophan) and variable quinones, such as ubiquinone and menaquinone in *Escherichia coli* and *Salmonella enterica*^29^. The respiratory quinone biosynthesis genes appeared to be enriched or depleted differently during the evolution of *Erwiniaceae* species under the natural selection to adapt to their hosts and environments for a long period of time. They exhibit remarkable genetic variation, often depending on the horizontal gene transfer of *car* operons on chromosomes and plasmids of *Pantoea* species^30^. The different gene clusters of chorimate synthetic enzymes (AroBEKAC), octaprenyl diphosphate synthetic enzymes (IspAB), ubiquinone synthetic enzymes (UbiABCDEFGHIJKX), isochorismate synthetic enzyme (EntC or MenF), menaquinone synthetic enzymes (MenABCDEHI), chromosomal and plasmid-encoded cartenoid synthetic enzymes (CrtE-(Idi)-CrtXYIB) , considered as chemotaxonomic markers of *Erwiniaceae* species, are depicted as colored boxes at the branch ends of the AAI tree (Fig. 3). Figure 4 shows a minimum evolution (ME) tree constructed using concatenated sequences of conserved AroBEKAC proteins. The branching patterns of AroBEKAC proteins are similar to those seen in the AAI tree. The AroBEKAC tree shows a broader evolutionary distance for the genus-level classification with type strains of *Erwiniaceae* at the rate of 0.175 amino acid substitution per site than the MLSA distance of 0.05 nucleotide substitution per site (see Supplementary Fig. S2). The *Erwiniaceae* clades showed often longer branch lengths, compared to other clades of *Enterobacteriaceae* and *Pectobactericeae*, or across the order* Enterobacteriales*^31^. *Erwiniaceae* strains include a large number of pathogens and symbionts with limited dispersal between the host populations. Symbionts, which can be classified into commensals and endosymbionts, tend to have reduced genome sizes as a consequence of losing genes whose functions depend on the hosts, as shown by parsimony analysis and sequence comparisons of signal peptides within the orthologous groups of *Enterobacteriales*^32^. Symbionts belonging to the genera *Buchnera*, *Phaseolibacter*, *Rosenbergiella*, *Tatumella,* and *Wigglesworthia* might have evolved from distinct lineages of *Erwiniaceae*. Their high evolution rate is thought to be caused by symbiotic function of some genes with a large number of substitutions or loss^33^. Our study demonstrated that AroBEKAC provided a reliable and effective molecular marker for the taxonomy and phylogenetic analysis of *Erwiniaceae* strains.

### Phenotypic characterization of strain PD-1

**Figure 5:**
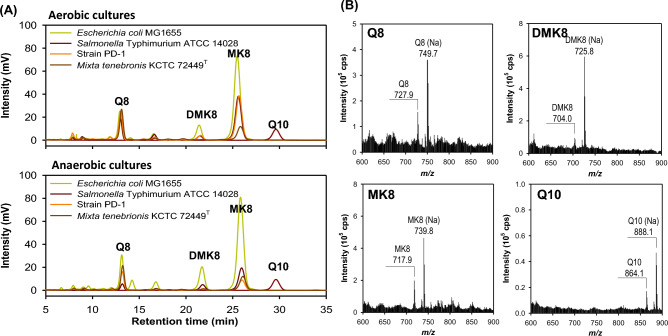
Analysis of respiratory quinones in aerobic and anaerobic culture conditions of strain PD-1 and reference strains. Cells cultivated in tryptic soy broth under aerobic and anaerobic conditions were used for extraction, purification, and quantification of the major ubiquinone 8 (Q8 [M-H]^+^ at *m/z* 728), demethylmenaquinone 8 (DMK8 [M-H]^+^ at *m/z* 704), and menaquinone 8 (MK8 [M-H]^+^ at *m/z* 718), detected by the reverse phase HPLC with UV detection at 254 nm and electrospray ionization-mass spectrometry in positive mode described in the Methods part and Supplementary Figure S5. As an exogeneous standard, ubiquinone 10 (Q10) was included.

Respiratory quinone compounds as chemotaxonomic markers of *Erwiniaceae* strains were analyzed by high-performance liquid chromatography with 254 nm UV detection coupled with electrospray ionization mass spectrometry. To compare differences between strain PD-1 and other strains, including four *Enterobacteriaceae* strains (*E. coli* K-12 MG1655, *S.* Typhimurium ATCC 14028, *Enterobacter pyrinus* KCTC 2590^T^, and *Entomohabitans teleogrylli* SCU-B244^T^) and five *Erwiniaceae* strains (*E. rhapontici* KACC 22740^T^, *M. tenebrionis* KCTC 72449^T^, *P. agglomerans* KACC 15275^T^, *P. ananatis* KACC 22739^T^, and *P. stewartii* KACC 22737^T^), molar ratio of quinone and menaquinone compounds in solvent extracted samples were determined by standard curves generated for each purified compound (Supplementary Fig. S5). Three major quinones detected in this study were assigned to ubiquinone 8 (Q8), demethylmenaquinone 8 (DMK8), and menaquinone 8 (MK8) (Fig. 5). The quinone composition of strain PD-1 was changed by decreasing the levels of DMK8 and MK8 compared to Q8 during the transition from aerobic to anaerobic conditions. These quinone profiles under aerobic and anaerobic conditions were more or less similar to those of *M. tenebrionis* KCTC 72449^T^ and *E. rhapontici* KCTC 22740^T^, but exhibited a negative correlation with the quinone profiles of the four *Enterobacteriaceae* strains (Table 2). The high level of Q8 in strain PD-1 implicates that it can play a key role in respiration utilizing oxygen and nitrate as electron acceptors in aerobic and anaerobic conditions, respectively, as does it in *E. coli*^29,34^. Interestingly, our analysis revealed multiple missing genes or defective genes such as UbiI, UbiK, and MenH in the genome of *S.* Typhimurium LT2^T^ compared to the genome of *S.* Typhimurium ATCC 14028. It has been observed that lateral transfer of genes is frequent in *S.* Typhimurim LT2, with 11% genes missing from the closely related *S. enterica* serovar Typhi and 29% missing from *E. coli* K12^35^. Plasmid taxonomic units in the order *Enterobacteriales* have been shown to exhibit a characteristic host distribution, frequently beyond the species barrier^36^. However, plasmid transmission and horizontal gene transfer is often constrained by taxonomic classification. For example, we found that three *Pantoea* species without menaquinone biosynthesis genes produced the only major Q8 and zeaxanthin, the characteristic yellow color produced by the plasmid and its associated genes in the *car* operon that may have been spread to various species of the *Pantoea*-*Duffyella*-*Mixta*-*Rosenbergiella* genera. As depicted in Fig. 3, some type strains of the genera *Pantoea* and *Duffyella* lost menaquinone, whereas some type strains of the genera *Mixta* and *Rosenbergiella* contain both menaquinone and cartenoid biosynthesis genes and operons. These strains appeared to gain or lose menaquinone at different rates after acquiring plasmid-associated *car* operons or vice versa. Using respiratory quinone and cartenoid markers, a high degree of genetic variation was found among the *Erwiniaceae* members.

**Table 2:** Molar ratio of respiratory quinones extracted from aerobically and anaerobically cultured cells of strain PD-1 and reference strains.

Strains	Q8	DMK8	MK8	Q8	DMK8	MK8	Correlation coefficient (*r*)
Strain PD-1	33.4 ± 1.4	21.8 ± 1.4	44.7 ± 0.8	55.3 ± 2.6	17.7 ± 2.8	26.9 ± 0.2	1.000
*Mixta tenebrionis* KCTC 72449^T^	70.3 ± 2.9	3.51 ± 1.9	26.2 ± 0.3	57.9 ± 2.5	13.4 ± 2.2	28.8 ± 0.3	0.619
*Erwinia rhapontici* KCTC 22740^T^	75.4 ± 3.2	6.39 ± 2.7	18.2 ± 0.02	54.3 ± 2.6	5.38 ± 3.6	40.3 ± 0.3	0.490
*Escherichia coli* K-12 MG1655	18.1 ± 0.8	35.5 ± 1.3	46.3 ± 0.9	17.3 ± 0.7	42.7 ± 1.4	40.0 ± 0.8	− 0.464
*Salmonella* Typhimurium ATCC 14028	27.4 ± 1.2	22.1 ± 1.5	50.5 ± 0.9	13.1 ± 0.8	45.9 ± 2.7	41.0 ± 0.6	− 0.347
*Enterobacter pyrinus* KCTC 2590^T^	34.7 ± 1.6	40.7 ± 2.4	24.5 ± 0.3	30.1 ± 1.4	48.6 ± 2.4	21.2 ± 0.3	− 0.567
*Entomohabitans teleogrylli* SCU-B244^T^	28.7 ± 1.4	59.7 ± 2.9	11.6 ± 0.04	18.2 ± 0.9	71.1 ± 2.7	10.7 ± 0.08	− 0.714
*Pantoea agglomerans* KACC 15275^T^	100	ND	ND	100	ND	ND	ND
*Pantoea ananatis* KACC 22739^T^	100	ND	ND	100	ND	ND	ND
*Pantoea stewartii* KACC 22737^T^	100	ND	ND	100	ND	ND	ND

Each strain was cultured in aerobic and anaerobic conditions of tryptic soy broth and PBS-washed cells were extracted using methanol-petroleum ether (1:1, v/v) for the quantitative and qualitative analysis of respiratory quinone compounds by HPLC with UV detection and mass spectrometry, as described in Supplementary Fig. S5.

*ND* not determined.

In order to differentiate the strain PD-1 from the nearest type strains, fatty acid methyl ester (FAME) analysis and API tests were carried out to compare with previously published data of *Erwiniaceae* strains. The FAME composition of strain PD-1 was similar to several strains from the genera *Mixta* and *Winslowiella*, (Pearson correlation coefficient *r* > 0.9), but differed from selected type species of the genera *Erwinia* and *Pantoea* (Table S1). The API 20E test results of strain PD-1 showed a high similarity (similarity index > 0.6) to those of *M. theicola* QC88-366^T^ and [*Pantoea*] *beijingensis* JZB2120001, when compared with closely related strains^24,37^ (Table S2). Strain PD-1 and all selected type strains of closely related species produced acids from D-glucose, D-mannitol, melibiose, and L-arabinose. When numerical data obtained from API 20E and 50CHB/E tests were examined among *Erwinia* members^24,38^, acid production from glycerol and erythritol was characteristic for strain PD-1 to be distinguished from the nearest relative [*Pantoea*] *beijingensis* JZB2120001 and other type strains of the genus *Erwinia* (Table S3). Table 3 summarizes characteristics of the strain PD-1 and closely related type strains of the genera *Winslowiella* and *Erwinia*, in which strain PD-1 is characteristically positive for acid production from glycerol and erythritol and is negative for esculin hydrolysis. Based on the phylogenetic and phenotypic characteristics, strain PD-1^T^ (= KCTC 13848BP^T^ = CGMCC 1.61905^T^) is proposed as the type strain of *Paramixta maurensis* gen. nov., sp. nov.

**Table 3:** Phenotypic characteristics for the differentiation of strain PD-1 and closely related strains^a^.

Test^b^	Strain PD-1		1	2	3	4
	24 h	48 h				
Citrate utilization	±	ND	–	–	+	–
Acetoin production	–	ND	–	+	–	–
Acid production from						
Glycerol	–	+	–	+	+	–
Erythritol	–	+	–	–	–	–
d-Arabinose	V	+	–	–	–	+
l-Xylose	–	–	–	+	+	–
Amygdalin	–	–	–	+	+	–
Arbutin	–	–	+	+	+	–
Esculin ferric citrate	–	–	+	+	+	+
Salicin	–	–	+	+	+	–
d-Cellobiose	–	–	–	+	+	–
d-Maltose	+	+	+	+	+	–
d-Lactose (bovine origin)	–	–	–	+	+	–
d-Melibiose	+	+	+	+	+	–
d-Saccharose (sucrose)	–	–	+	+	+	–
d-Raffinose	–	–	–	+	–	–
Gentiobiose	+	V	+	+	–	–
d-Lyxose	–	–	+	–	–	–
d-Fucose	V	+	–	+	+	–
d-Arabitol	+	+	–	+	+	+
Potassium gluconate	–	–	–	V	–	+
Potassium 2-keto-gluconate	–	–	–	–	–	+

*V* variable, *ND* not determined.

^a^Data taken from Liu et al.^24^ and Campillo et al.^38^: **1,** [*Pantoea*] *beijingensis* JZB2120001; **2,** *W. iniecta* B120^T^; **3,** *W. toletana* CFBP 6631^T^; **4,** *E. oleae* CFBP 8201^T^.

^b^Numerical analysis of strain PD-1 was determined using API 20E and 50CHB/E tests as shown in Tables S2and S3.

### Polyol pathway

**Figure 6:**
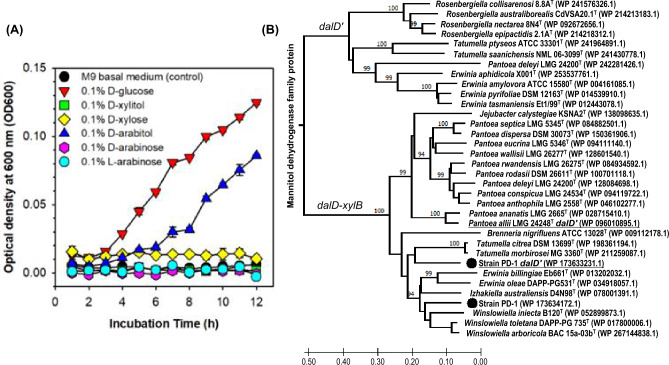
Utilization of D-arabitol substrate and the evolutionary relationship of D-arabitol dehydrogenase. (**A**) Growth curves of strain PD-1 with D-glucose, D-xylitol, D-xylose, D-arabitol, D-arabinose, and L-arabinose in M9 minimal medium. (**B**) Minimum evolution phylogenetic tree of D-arabitol dehydrogenases (DalD) in strain PD-1 and closely related strains. The optimal tree is shown with more than 90% of 1000 replicate trees in the bootstrap test and drawn to scale, as the units of the number of amino acid substitutions per site, computed using the Dayhoff matrix based method in MEGA X. There was a total of 455 positions in the final dataset of 35 amino acid sequences aligned using MAFFT with BLOSUM62 matrix.

Strain PD-1 was able to utilize d-arabitol and glucose as the sole carbon and energy source, but it cannot grow on M9 minimal medium containing d-xylitol, d-xylose, d-arabinose, and L-arabinose (Fig. 6A). The genome analysis revealed that strain PD-1 contains two homologous genes coding for d-arabitol 4-dehydrogenases DalD (WP_173634172.1 and WP_173633231.1) categorized as mannitol dehydrogenase family protein. However, any of the homologous gene and the capability of utilizing D-arabitol was not detected from [*Pantoea*] *beijingensis* JZB2120001. The genome of strain PD-1 contains a gene set encoding DalD, D-xylulokinase XylB (synonym, AtlK), and ribulose-phosphate 3-epimerase for d-arabitol catabolism via d-xylulose-5-phosphate and d-ribulose-5-phosphate in the pentose phosphate pathway (Table 4). The strain PD-1 carries the complete gene sets for glycolysis via Embden-Meyerhof-Parnas pathway, pentose phosphate pathway, TCA cycle, and different pathways that are relevant for the utilization of d/l-fucose, d-galactose, d-mannose, and d-mannitol, except for L-rhamnose.

**Table 4:** Proteins for the degradation of monosaccharides derived from hemicellulose.

Enzymes	Gene ID (locus tag)	Homolog ID (UniProtKB)	Homolog ID (NCBI)	Identity (%)	E-values	References
Utilization of d-arabitol						^39^
d-arabinitol 4-dehydrogenase	PMPD1_2270	UPI0003456E87	WP_017800006.1	83.4	0	
d-xylulokinase	PMPD1_2271	UPI0005526436	WP_034914917.1	81.2	0	
Ribulose-phosphate 3-epimerase	PMPD1_0329	D4I319	WP_004160688.1	91.2	2.99E–152	
Utilization of l-fucose						^40^
l-fucose dehydrogenase	PMPD1_3766	A0A085G0Q3	WP_034796198.1	76.7	1.03E–131	
l-fuconolactonase	PMPD1_3767	K8A015	WP_007671895.1	64.0	4.22E–115	
l-fuconate dehydratase	PMPD1_3768	A0A0H3FNB4	WP_015703671.1	85.4	0	
2-keto-3-deoxy-l-fuconate dehydrogenase	PMPD1_1355	A0A419N4K2	RJT39839.1	81.9	1.56E–145	
l-2,4-diketo-3-deoxyrhamnonate hydrolas	PMPD1_1354	A0A419N4D8	RJT39838.1	81.4	4.73E–170	
l-lactate dehydrogenase (cytochrome)	PMPD1_1350	Q6DAY3	WP_011091762.1	89.3	0	
Utilization of d-galactose						^41^
d-galactose 1-epimerase	PMPD1_1476	UPI00034927D0	WP_026111404.1	77.0	0	
Galactokinase	PMPD1_1477	A0A2J9E736	WP_033789052.1	89.7	0	
Galactose-1-phosphate uridylyltransferase	PMPD1_1478	A0A0L7TC12	WP_052896837.1	88.8	0	
UTP–glucose-1-phosphate uridylyltransferase	PMPD1_2840	A0A0L7T9U5	WP_052897567.1	90.3	0	
Phosphoglucomutase	PMPD1_1435	H3RAQ5	WP_006118435.1	92.1	0	
Utilization of d-mannose						^42^
Mannose PTS system EIID component	PMPD1_2423	A0A126VIS9	WP_015672722.1	91.5	0	
Mannose PTS system EIIC component	PMPD1_2422	P69803	NP_310555.1	89.1	2.56E–148	
Mannose PTS system EIIAB component	PMPD1_2421	A0A1X1DVK0	AUX93705.1	88.7	0	
Mannose-6-phosphate isomerase	PMPD1_2150	UPI00036A8424	WP_017802255.1	80.3	0	
Utilization of d-mannitol						^43^
Mannitol PTS system EIICBA	PMPD1_0116	D8MLM5	WP_013204522.1	87.9	0	
Mannitol-1-phosphate 5-dehydrogenase	PMPD1_0115	A0A0L7TDB6	WP_052898197.1	90.0	0	
Utilization of d-glucuronate						^44^
Glucuronate isomerase	PMPD1_3844	A0A0L7SVZ1	WP_052902905.1	87.2	0	
Fructuronate reductase	PMPD1_2875	A0A0U5L796	WP_067432173.1	78.8	0	
Mannonate dehydratase	PMPD1_2872	C6D9S0	WP_012773497.1	91.8	0	
2-dehydro-3-deoxygluconokinase	PMPD1_0039	A0A0L7SYX4	WP_052901041.1	84.9	0	
2-dehydro-3-deoxyphosphogluconate aldolase (duplicate)	PMPD1_2534	H3RFN6	WP_006120281.1	89.2	1.91E–133	
	PMPD1_0571	A0A0A3YQQ3	WP_034896908.1	79.6	5.18E–115	

The ME phylogenetic tree shows that two DalD proteins of strain PD-1 have evolved from different *dalD* genes in *Erwiniaceae* strains (Fig. 6B). Similarly, *P. deleyi* LMG 24200^T^ possessed two *dalD* genes that have evolved at different rates. The different *dalD* genes on chromosomes showed significant differences in gene arrangement between two clades. One clade, which contained *T. ptyseos* and *T. saanichensis*, comprised single *dalDʹ* genes. The other clade containing *T. citrea* and *T. morbirosei* shared mostly *dalD*-*xylB* operon structures, except for the single *dalDʹ* genes of strain PD-1 and *P. allii* LMG 24248^T^. The *dalD-xylB* genes appear to be phylogenetically conserved in a part of *Erwiniaceae* strains, such that closely related strains share a similar trait. In this context, strain PD-1 and type strains of the genus *Winslowiella*, which contain the conserved *dalD-xylB* genes, are capable of utilizing D-arabitol, but [*Pantoea*] *beijingensis* JZB2120001 without any *dalD* gene cannot utilize it (see Table 3). Because D-arabitol is naturally present in lichens, certain mushrooms, some plants and seeds, genes for D-arabitol catabolism have been suggested to be a model for studying the evolution of enzyme pathways in the *Enterobacteriaceae-Erwiniaceae* members^45,46^. However, because the genetic variation of DalD was not determined in many bacteria, including the genera *Duffyella*, *Mixta*, *Phaseolibacter*, *Wigglesworthia*, and *Buchnera*, it could explain the evolutionary history of a set of *Erwiniaceae* strains, but not all strains. This analysis suggests that DalD may have evolved as a specific trait of some *Erwiniaceae* strains for so long that they were substantially differentiated and specialized to particular hosts or niches. These strains may have evolved DalD to degrade D-arabitol and have undergone gain or loss of function to utilize new substrates. Besides, strain PD-1 possesses various glycosyl hydrolases that are involved in degradation of polysaccharides, mainly composed of glucose and galactose (Table S4). These genetic findings are in line with the results of the substrate utilization tests, which suggest that strain PD-1 may play a role in the degradation of a variety of carbohydrates and polysaccharides derived from fungi and plants.

### Auxin analysis

Some strains in the genera *Erwinia* and *Pantoea* have been reported to interact with plants by producing a natural plant hormone auxin, indole-3-acetic acid (IAA), by different pathways via indole-3-pyruvic acid or/and indole-3-acetamide^47–49^. IAA biosynthesis is common among plant-associated bacteria that develop symbiotic relationships ranging from pathogenesis to mutualism^50^. We analyzed that exponential-phase cells of strain PD-1 were able to produce a high concentration of IAA at ~ 0.4 mg/L, determined by LC–MS/MS using authentic chemical as standard (Table 5). The LC/electrospray ionization mass spectra (ESI–MS) of the precursor ion [M + H]^+^ of IAA at *m/z* 176.1 and its product ion at *m/z* 130.1 were used for the determination of IAA concentration within a linear range of 7.81–125 g/mL (Supplementary Fig. S6). The concentration of IAA routinely produced by strain PD-1 in the TS broth was considerably (one or two magnitude of order) higher than those of other IAA-producing *Klebsiella* and *Bacillus* strains and 2 ~ 5 times lower than IAA production of *P. agglomerans* strain PVM in the optimal medium^51^. However, no intermediates for the IAA biosynthesis pathways such as indole-3-pyruvic acid, indole-3-acetamide, and tryptamine were detected in this study. Genome analysis showed that strain PD-1 contains the genes coding for indole-3-pyruvate decarboxylase (locus tag PMPD1_3174) and aldehyde dehydrogenase (PMPD1_0173), which can synthesize IAA via the indole-3-pyruvic acid pathway, as similarly did *Bradyrhizobium elkanii*^52^. These enzymes have been broadly found in members of the order *Enterobacteriales*. Except for this pathway, strain PD-1 possesses no alternative pathway for auxin formation via indole-3-acetamide and tryptamine intermediates produced by tryptophan monooxidase and decarboxylase, respectively. Besides, strain PD-1 has a gene set (operon) of PqqBCDEF (PMPD1_0223–PMPD1_0227) for the biosynthesis of pyrroloquinoline quinone (PQQ) which is a plant growth promotion factor of rhizobacteria^53^. These findings suggest that strain PD-1 has a potential of interacting with plants as well as playing a role in compost.

**Table 5:** Quantification of indole-3-acetic acid produced by strain PD-1.

Chemical name	Linear detection range (ng/mL)	Calibration curve	*R*^2^	IAA concentration (mg/L)
Indole-3-acetic acid (IAA)	7.81–125	Y = 566488x + 2302.2	0.9962	0.409 ± 0.002

## Methods

### Strains and culture conditions

Strain PD-1 was isolated from mushroom (*P. eryngii*) compost obtained from Hwasun-gun (34.99939 N, 126.91040 E), Jeonnam Province, South Korea. The composted waste of *P. eryngii* culture substrate, consisting of 70–80%(w/w) oak sawdust and 20 ~ 30%(w/w) rice/wheat gran, was collected in a 50 mL sterile tube and transported on the same day to the research laboratory at Jeonnam National University. One gram (wet weight) of the collected sample was taken and homogenized in 100 mL of sterilized water at 25 °C and 150 rpm for 24 h. After brief centrifugation at (4,200 × g) for 2 min, 200 μl of the supernatant was spread on a Luria Bertani (LB) agar plate (BD, Sparks MD, USA). The plate was incubated at 37 °C for 48 h to isolate the single colony of the dominant morphology. As reference strains, *E. coli* K-12 MG1655, *S.* Typhimurium ATCC 14,028, *E. pyrinus* KCTC 2590^T^, *E. teleogrylli* SCU-B244^T^, *E. rhapontici* KACC 22740^T^, *M. tenebrionis* KCTC 72449^T^, *P. agglomerans* KACC 15275^T^, *P. ananatis* KACC 22739^T^, and *P. stewartii* KACC 22737^T^ were obtained from Korean Collection for Type Cultures (KCTC) and Korean Agricultural Culture Collection (KACC). The isolate and reference strains were routinely cultured on BD tryptic soy (TS) broth and R2A medium to prepare 80% (v/v) glycerol stocks stored at − 80 °C.

### DNA analysis

Genomic DNA of bacterial cells was isolated using a MG Genomic DNA Purification Kit (MGmed, Korea). A partial 16S rRNA sequence was prelimarily amplified by PCR with the universal primers, 27F (5′-AGAGTTTGATCMTGGCTCAG-3′) and 1492R (5′-TACGGYTACCTTGTTACGACTT-3′), and sequenced by Sanger sequencing. The 16S rRNA gene pairwise similarity was calculated using the EzBioCloud database and tools^54^. For genomic DNA sequencing using the PacBio RS II system (Pacific Biosciences, Menlo Park CA, USA), a high-quality DNA greater than 40 kb was prepared using AMPure PB magnetic beads (Beckman Coulter Inc., Brea, CA, USA). Utilizing a NanoDrop spectrophotometer and a Qubit fluorometer, genomic DNA was quantified, and to check its quality, 200 ng/μl of the DNA extract was run on a field-inversion gel. The PacBio DNA Template Prep Kit 1.0 was used to prepare a 10-μl DNA library, and the PacBio DNA/Polymerase Binding Kit P6 was used to anneal the SMRTbell templates. Sequencing runs with C4 chemistry and 240 min movies were performed with the PacBio DNA Sequencing Kit 4.0 and SMRT Cell 8 M. Whole genome sequencing of the strain PD-1 was performed using the Illumina HiSeq. The hierarchical genome assembly process HGAP3 was used to assemble the raw data^55^ and their annotation was conducted by Rapid Annotation using Subsystem Technology (RAST) server^56^. In silico DNA-DNA hybridization (DDH) and the average nucleotide identity (ANI) were used to obtain an estimate of the overall similarity between two genome sequences^57–59^. The whole genome sequence of strain PD-1 has been deposited in NCBI under the GenBank accession numbers, CP054212 for the chromosome and CP054213 for the plasmid.

### Phenotypic analysis

Cell morphology was checked using a light microscope and a Zeiss field emission scanning electron microscope (FE-SEM)-II Gemini 500 + EDS (Oxford) at 125 eV. Gram staining of bacteria was performed using a standard method^60^. In order to determine an optimal growth condition, we examined growth rates of cells at various temperatures (10, 15, 20, 25, 30, 35, and 40 °C), pH, and salt concentrations in R2A agar plates and broth. Numerical analyses were performed using API 20E and API 50 CHB/E test kits according to the manufacturer instructions (bioMérieux, Marcy ľEtoile, France).

### Fatty acid methyl ester analysis

Fatty acid methyl ester (FAME) samples of cells grown in TS broth were prepared, as described previously^61^, and analyzed on a gas chromatography (GC)-mass spectrometer (Shimadzu GC-17A) equipped with a Supelco SP-2560 capillary GC column. The following conditions applied to the GC: the initial temperature was held at 100 °C for 5 min, then increased at 3.5 °C/min up to 240 °C, and held for 30 min. FAME components were identified using a FAME standard mixture (Sigma-Aldrich, cat# 1269119).

### HPLC–UV/mass spectrometry for detection of respiratory quinone and auxin

Exponentially growing cells of strain PD-1 and reference strains in TS broth were centrifuged at 4000 rpm (3515×*g*) for 10 min. The decant supernatant (10 mL) of strain PD-1 was mixed thoroughly with 10 mL ACN, 1 g NaCl, and 4 g MgSO_4_ anhydrous for 3 min to extract auxin. The sample was centrifuged at 4000 *rpm* for 5 min and the supernatant (8 mL) was agitated vigorously with 0.4 g octadecane (Agilent, USA) and 1.2 g MgSO_4_ for 1 min. After centrifugation at 4000 *rpm* for 5 min, the separated organic layer (2 mL) was concentrated using nitrogen drying. The extract was dissolved in 200 μl 100% methanol (MeOH) containing 0.1% formic acid and analyzed on a Shimadzu LC-10ADvp system (Shimadzu, Japan) coupled to an API2000 mass spectrometer (AB SCIEX, Framingham, MA, USA). The column was ZORBAX C_18_ (4.6 × 250 mm, 5-µm particle size, Agilent, Santa Clara CA, USA). The mobile phases, which were composed of water containing 2 mM ammonium formate with 0.1% formic acid (mobile phase A) and MeOH with 0.1% formic acid (mobile phase B), were pumped at a flow rate of 1 mL/min. The chromatographic condition was a 10-min linear gradient of 0–100% B, then held at 100% B for 5 min, and equilibrated for 15 min at 0% B before the next run. A column used in this study was calibrated using an authentic indole-3-acetic acid (IAA) sample and the concentration range of 7.81–125 ng/mL was used to construct the standard curve with the injection volume of 20 µl. The limit of detection (LOD) and limit of quantification (LOQ) of IAA with mass spectrometry were determined at threefold and tenfold signal-to-noise (S/N) ratios, respectively. The detection and quantification of IAA was performed using selected reaction monitoring (SRM) of characteristic transition ions Q1 and Q3 in positive mode, the maximum sensitivity of which was evaluated with focusing potential (360.0 V), decluttering potential (21.0 V), collision energy (31.0 eV), collision cell entrance potential (28.0 V), entrance potential (8.0 V), and collision cell exit potential (8.0 V). Conditions for mass spectrometry included curtain gas at 30 psi, source temperature at 500 °C, spray voltage at 5500 V, and ion source gas at 50 psi. To analyze respiratory quinones, harvested cells of strain PD-1 and nine reference strains were inoculated to make the optical density of 1.0 at 600 nm in each tube containing 10 mL TS broth under aerobic and anaerobic conditions. The anaerobic condition was maintained in a glove box (Coy Labs, Grass Lake, MI, USA) filled with a mixture of 5% H_2_/10% CO_2_/85% N_2_ by volume. The culture tubes were tightly sealed and incubated with shaking (200 rpm) for 24 h at 37 °C for *E. coli* and *S.* Typhimurium and at 30 °C for strain PD-1 and the other strains. Cells were centrifuged at 4,000 rpm for 10 min and the pelleted cells were vigorously washed with deionized water. After centrifugation at 4000 rpm for 10 min, wet weight of harvested cells was measured and converted to a volume to mix with 9 volumes of methanol-petroleum ether (1:1, v/v) for respiratory quinone extraction. After vigorous vortex and sonification for 10 min, the upper layer of petroleum ether was decanted into a new tube, dried in vacuo, and dissolved in 200 μl 100% ethanol to analyze on a Shimadzu LC-10ADvp system (Shimadzu, Japan) equipped with a UV detector at 254 nm and an API2000 mass spectrometer (AB SCIEX, Framingham, MA, USA). The HPLC column was Inertsil ODS-3 V (4.6 × 150 mm, 5-µm particle size, GL Sciences, Tokyo, Japan), heated at 53 °C. The solvent was anhydrous MeOH containing 0.5% formic acid, pumped at a flow rate of 1 mL/min. An internal standard, 100 μg/mL ubiquinone 10 (Q10), was included in the sample analysis. The ESI–MS analysis was operated in positive mode under the same conditions as above. *E. coli* MG1655 cells aerobically grown in 1 L TS broth were used to purify and determine molar concentrations of isolated quinone compounds (Q8, DMK8, and MK8) by repeated chromatography on ZORBAX ODS column (9.4 × 250 mm) and Waters Nova-Pak C18 column (3.9 × 150 mm) using methanol as eluent.

### Statistical analysis

Bacterial culture experiments were performed at least three independently, and the results were reported as means and standard deviations of the means. Clustal Omega was used to carry out multiple sequence alignment for MLSA and phylogenetic analysis^62^. Amino sequences of DalD proteins were aligned using MAFFT with BLOSUM62 matrix^63^. Evolutionary analyses of aligned nucleotides and amino acid sequences were performed using the Neighbor-Joining method and Minimum Evolution method, respectively, in MEGA X^64^.

### Supplementary Information

Supplementary Information.

Supplementary Data.

## Data Availability

All data generated or analyzed during this study are included in this published article.
