# Author Correction: *Paramixta manurensis* gen. nov., sp. nov., a novel member of the family *Erwiniaceae* producing indole-3-acetic acid isolated from mushroom compost

**DOI:** 10.1038/s41598-024-68518-0

**Published:** 2024-08-13

**Authors:** Jueun Kim, Hyosuk Yun, Aminallah Tahmasebi, Jiyoung Nam, Ha Pham, Yong‑Hak Kim, Hye Jung Min, Chul Won Lee

**Affiliations:** 1 Department of Chemistry, Chonnam National University, Gwangju, 61186 Republic of Korea; 2 Research Center, DAESANG InnoPark, Gangseo-gu, Seoul, 07789 Republic of Korea; 3 Department of Agriculture, Minab Higher Education Center, University of Hormozgan, Bandar Abbas, Iran; 4 Institute of Well-Aging Medicare & CSU G-LAMP Project Group, Chosun University, Gwangju, 61452 Republic of Korea; 5 Department of Microbiology, Daegu Catholic University School of Medicine, Daegu, 42472 Republic of Korea; 6 Department of Cosmetic Science, Gwangju Women’s University, Gwangju, 62396 Republic of Korea

Correction to: *Scientific Reports* 10.1038/s41598-024-65803-w, published online 05 July 2024

In the original version of this Article a previous rendition of Figures 1, 3, 4, 5, 6 was published. As a result, their legends were incorrect.

The original Figures 1, 3, 4, 5, 6 and their accompanying legends appear below.

**Figure 1:**
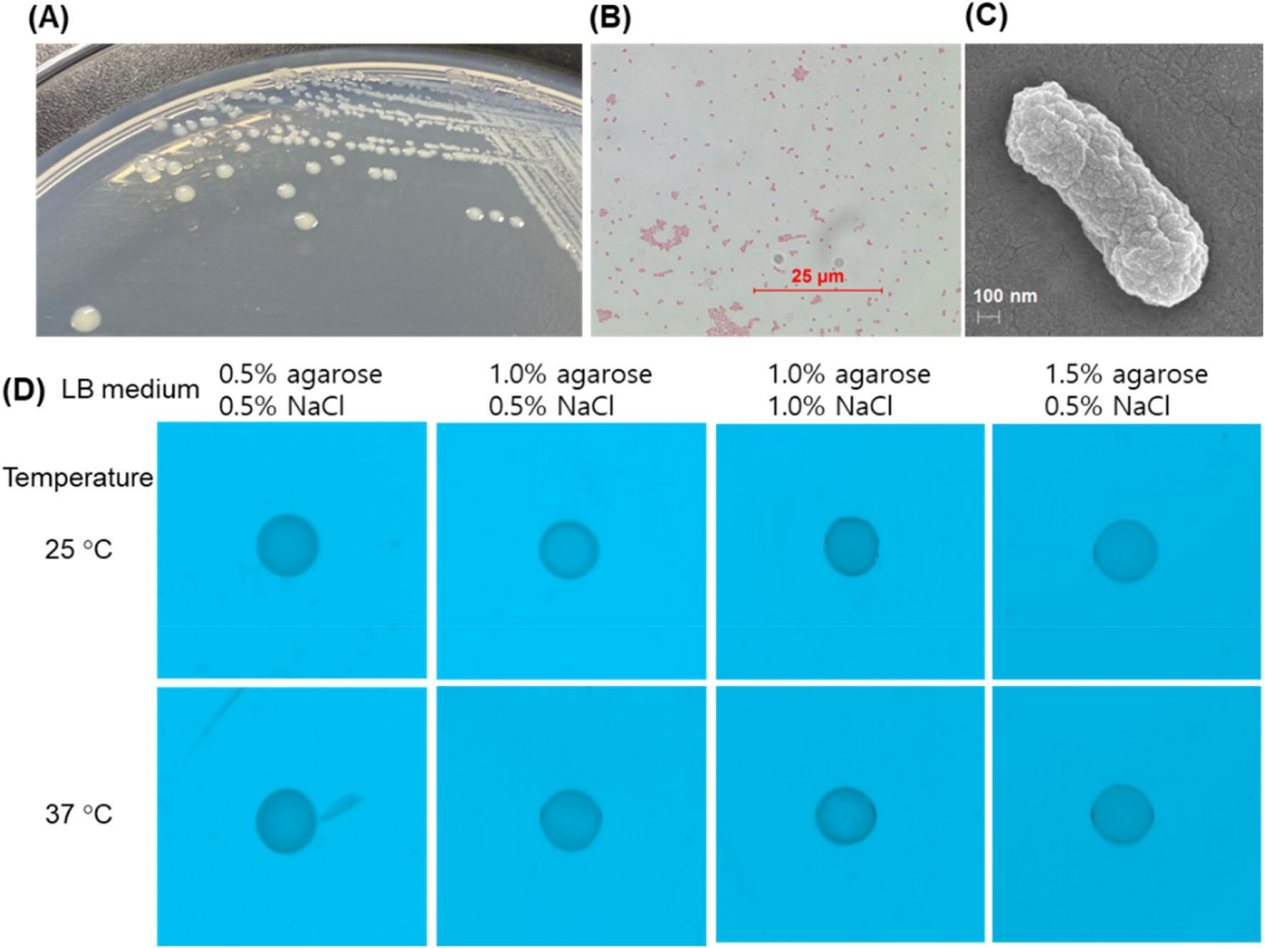
Morphology and motility test of strain PD-1. (**A**) Morphology of colonies on a R2A plate. (**B**) Gram-negative cells at ×1000 magnification under a light microscope. (**C**) Scanning electron microscopy (SEM) of gold-coated cells. (**D**) No motility of cells measured at 25 °C and 37 °C after spotting of a culture aliquot (5 μL) on LB media with different concentrations of Bacto agarose (0.5, 1.0, and 1.5%, w/v) and NaCl (0.5 and 1.0%, w/v).

**Figure 3:**
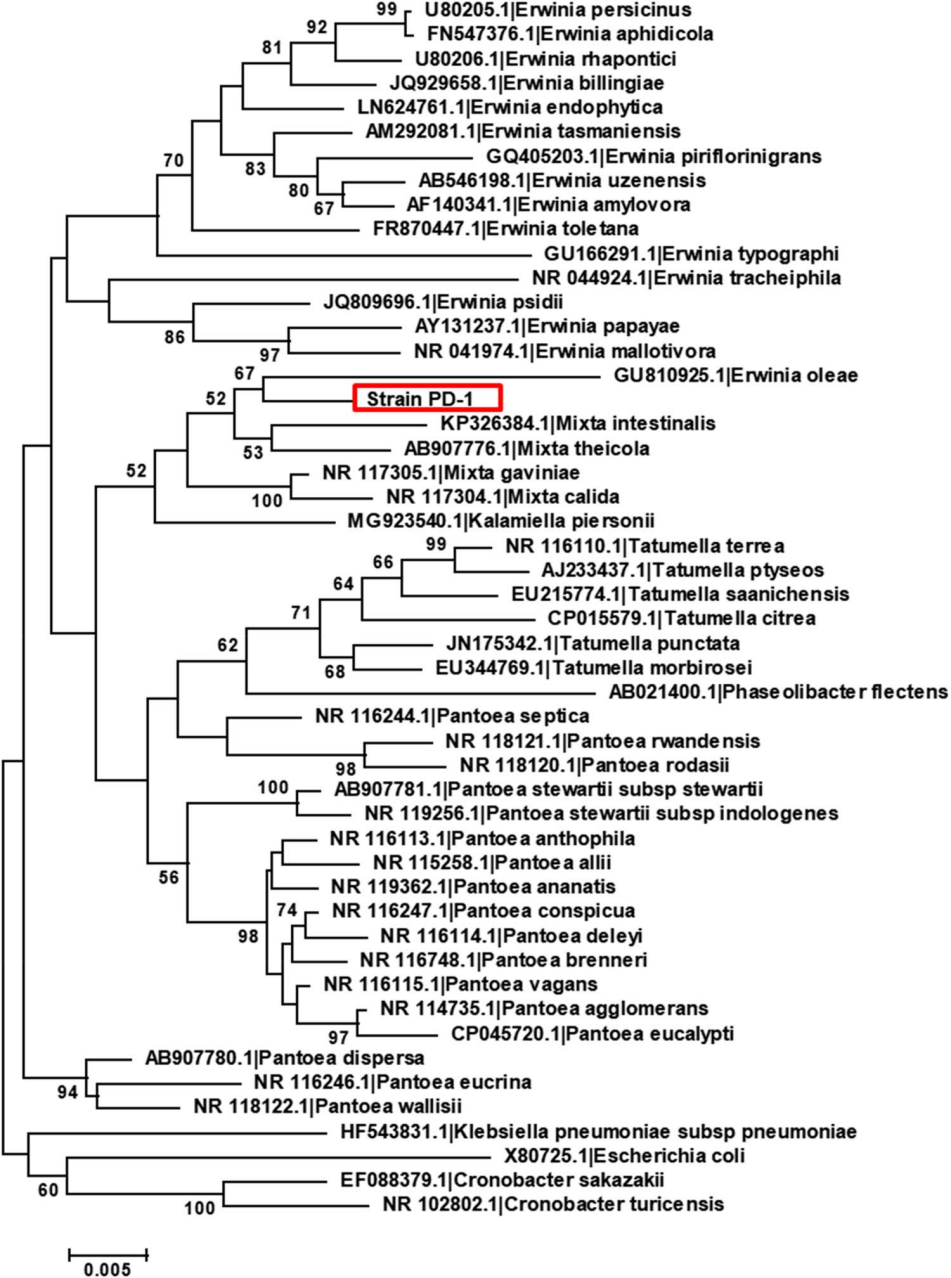
Phylogenetic analysis of 16S rRNA genes in strain PD-1 and related taxa. A neighbor-joining tree of the 16S rRNA gene is shown with the optimal tree (sum of branch length = 0.51196467). More than 50% of replicate trees in which the associated taxa clustered together in the bootstrap test (1000 replicates) are shown next to the branches. The scale is drawn with branch lengths in the unit of the number of base substitutions per site as the evolutionary distance computed by the Maximum Composite Likelihood method. The analysis involved 50 nucleotide sequences. All ambiguous positions were removed for each sequence pair. There were a total of 1287 positions in the final dataset. Evolutionary analyses were conducted in MEGA7.

**Figure 4:**
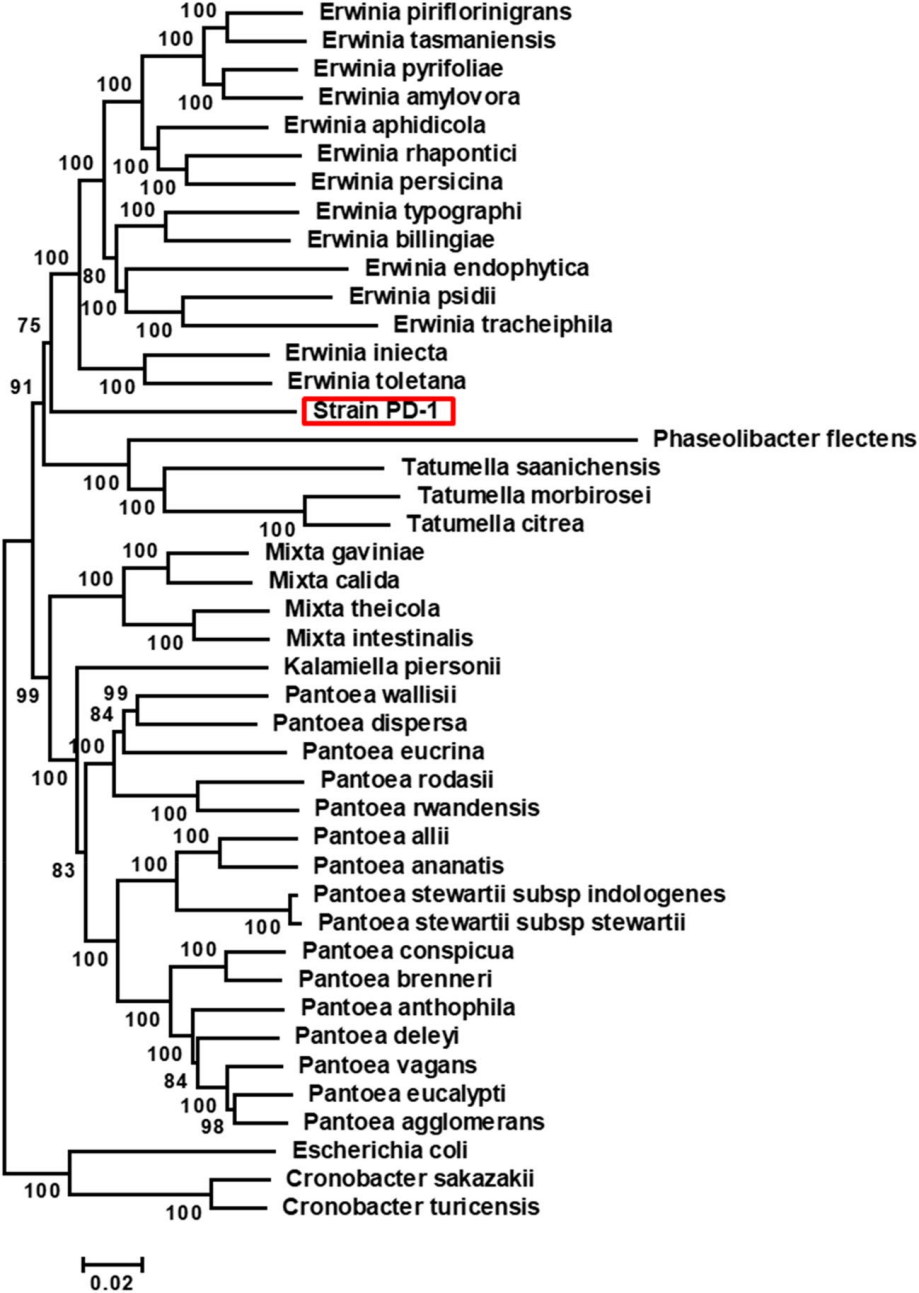
Multilocus sequence analysis of strain PD-1 and related taxa. A neighbor-joining tree of the concatenated core gene set (*atpD-carA-gyrB-infB-rpoB-recA*) is shown with the optimal tree (sum of branch length = 2.23121016). The unpaired nucleotides at the 5′ and 3′ ends of each gene were trimmed based on CLUSTAL W multiple alignment. More than 50% of replicate trees in which the associated taxa clustered together in the bootstrap test (1000 replicates) are shown next to the branches. The scale is shown with branch lengths in the units of the number of base substitutions per site as the evolutionary distances computed by the Maximum Composite Likelihood method. The analysis involved 43 nucleotide sequences. Codon positions included were 1st + 2nd + 3rd. All ambiguous positions were removed for each sequence pair. There was a total of 12,813 positions in the final dataset. Evolutionary analyses were conducted in MEGA7.

**Figure 5:**
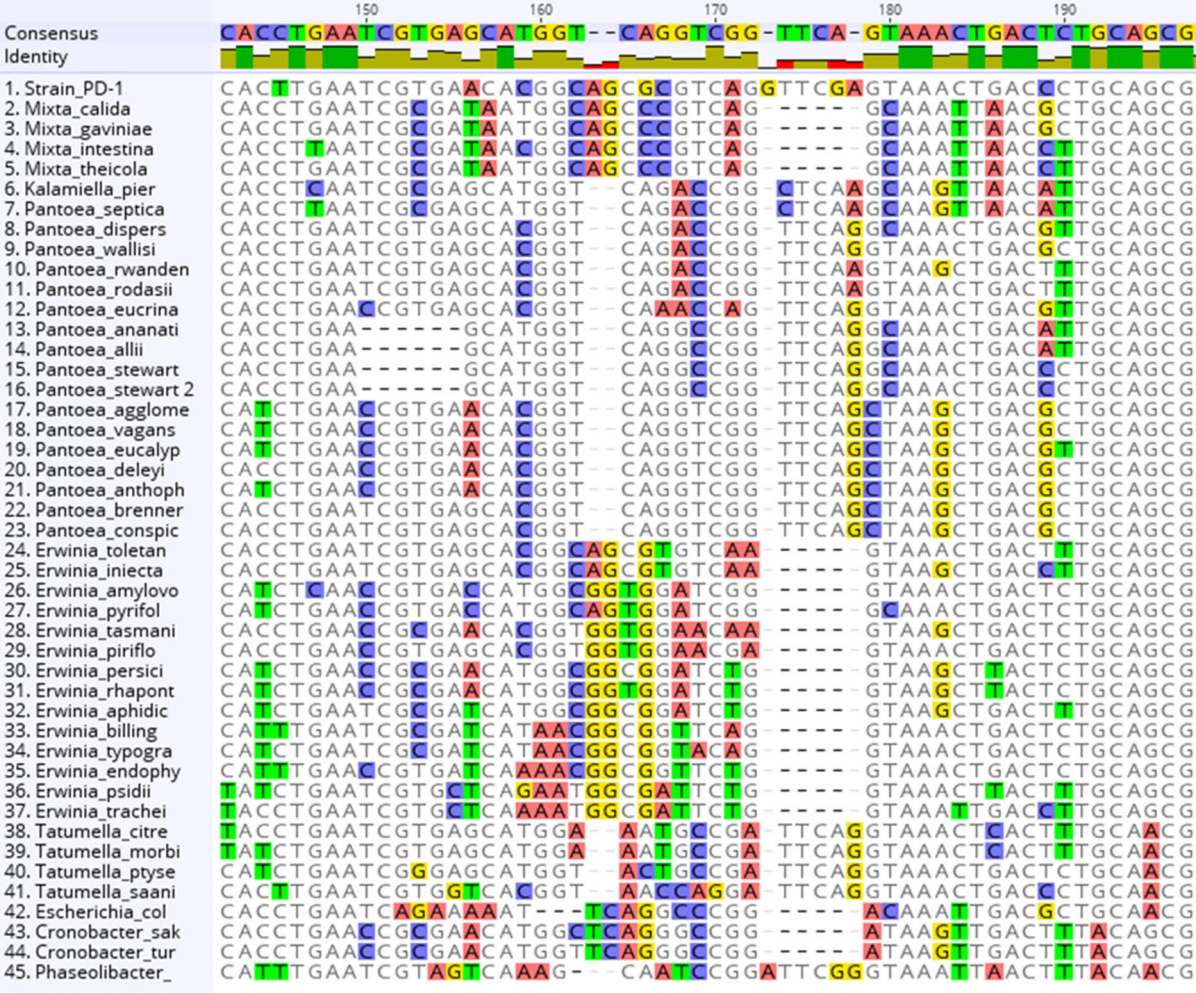
Genus-specific InDel pattern of infB. There was a genus-specific InDel pattern in the aligned *infB* sequences. Strain PD-1 had a large difference in genomic DNA with high rates of single nucleotide polymorphisms and insertion/deletion (InDel) mutations as well as novel loci, likely plasmid borne, which probably influence population differentiation against selection pressure in specialized niches.

**Figure 6:**
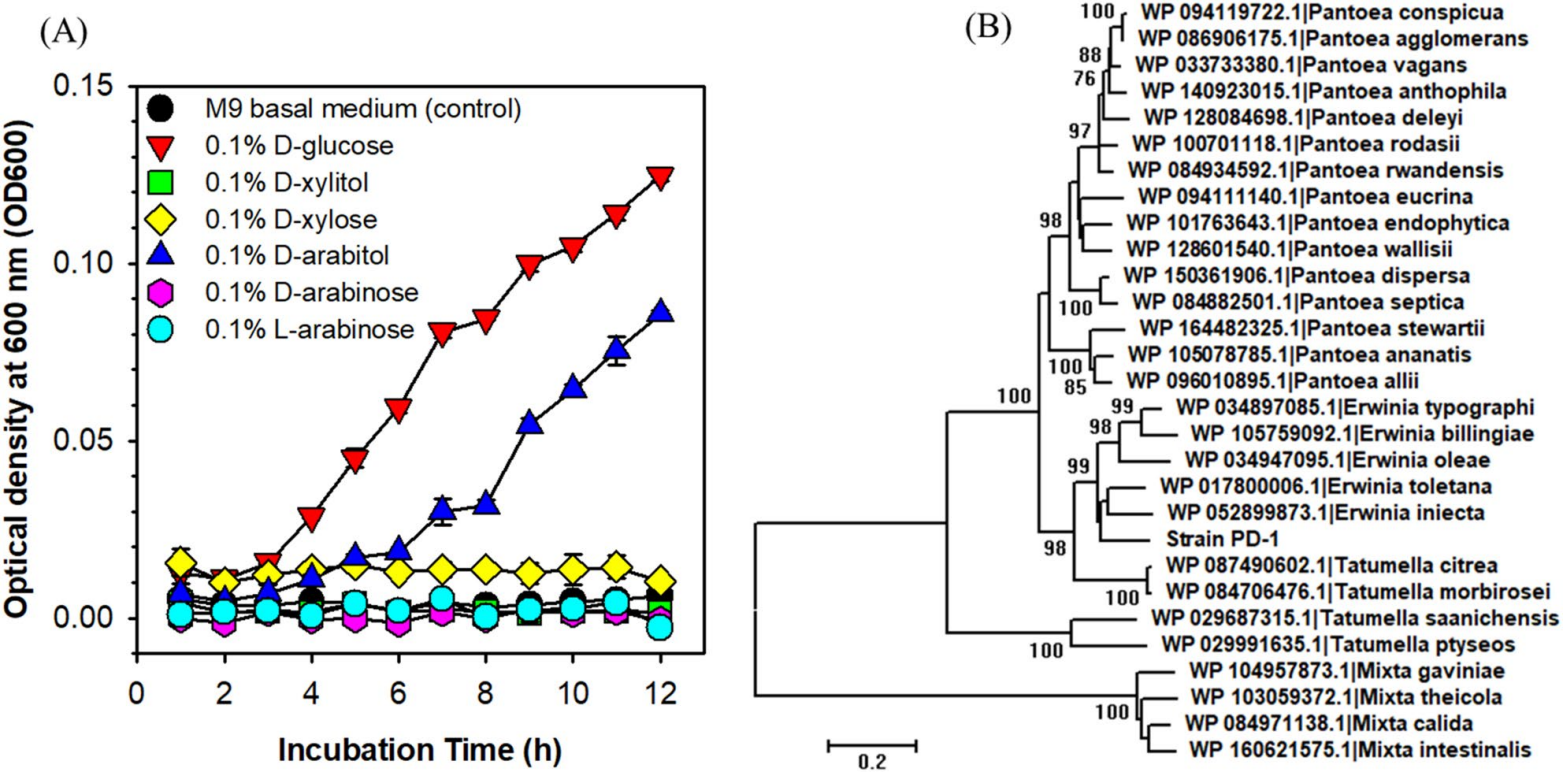
Utilization of d-arabitol substrate and the evolutionary relationship of d-arabitol dehydrogenase. (**A**) Growth curves of strain PD-1 with d-glucose, d-xylitol, d-xylose, d-arabitol, d-arabinose, and l-arabinose in M9 minimal medium. (**B**) Phylogenetic analysis of d-arabitol dehydrogenases in strain PD-1 and closely related strains involving a total of 495 positions of 29 amino acid sequences aligned using MAFFT with BLOSUM62 matrix. The optimal tree with the sum of branch length = 4.38424399 is shown with more than70% of replicate trees in the bootstrap test (1000 replicates) by the minimum evolution (ME) method. The scale shows the evolutionary distance determined as the number of amino acid substitutions per site by the Dayhoff matrix- based method. The ME tree was searched using the close-neighbor-interchange algorithm at a search level of 1 in MEGA7. All ambiguous positions were removed for each sequence pair.

Additionally, the Supplementary data file was omitted from the Supplementary Information section.

The original Article has been corrected and The Supplementary data file now accompanies the Article.

### Supplementary Information

Supplementary Information.

