# Supplementary material for: Paramixta manurensis gen. nov., sp. nov., a novel member of the family Erwiniaceae producing indole-3-acetic acid isolated from mushroom compost
Source: Sci Rep. 2024 Jul 5;14:15542. doi: 10.1038/s41598-024-65803-w (PMC11226699; doi:10.1038/s41598-024-65803-w)
Supplement: Supplementary file 1 — Supplementary Information. [file 41598_2024_65803_MOESM1_ESM.docx]

**Supplementary Materials:**

**Table S1.** Fatty acid methyl ester profiles for the differentiation of strain PD-1 from the closely related genera *Erwinia,* *Mixta*, *Pantoea*, and *Winslowiella*.

| **Fatty acid components*^a^*** | **1** | **2** | **3** | **4** | **5** | **6** | **7** | **8** | **9** | **10** | **11** | **12** | **13** |
| --- | --- | --- | --- | --- | --- | --- | --- | --- | --- | --- | --- | --- | --- |
| C_12 : 0_ | 4.2 | 4.2 | 5.9 | 9.2 | 2.5 | 4.1 | 7.7 | 8.8 | - | 3.8 | 5.4 | 3.2 | 4.6 |
| C_12 : 0_ 3OH | 0.3 | - | - | - | - | - | - |  | - | - | - | - | - |
| C_14 : 0_ | 5.6 | 6.5 | 8.4 | - | 8.2 | 5.7 | - | 6.3 | - | 3.1 | 5.4 | 4.9 | - |
| C_16 : 1_ ω5*c* | 0.1 | - | - | - | - | - | - |  | - | - | - | - | - |
| C_16 : 0_ | 38.8 | 34.9 | 32.5 | 29.7 | 35.7 | 29.8 | 33.9 | 25.3 | 32.0 | 33.8 | 30.3 | 30.5 | 26.8 |
| C_17 : 0_ iso | 0.3 | - | - | - | - | - | - |  | - | - | - | - | - |
| C_17 : 1_ ω7*c* | 0.3 | - | - | - | - | - | - |  | - | - | - | - | - |
| C_17 : 0_ cyclo | 13.4 | 15.2 | 10.8 | 14.0 | 19.5 | 13.8 | 7.3 | 11.9 | 10.3 | 3.9 | 3.8 | 8.5 | 3.0 |
| C_17 : 0_ | 0.3 | 0.4 | - | 2.4 | - | - | 2.0 |  | - | 0.4 | 0.8 | - | 1.9 |
| C_18 : 3_ ω6*c* (6, 9, 12)*_­_* | 0.2 | - | - | - | - | - | - |  | - | - | - | - | - |
| C_18 : 0_ | 0.4 | 1.0 | 1.2 | - | 0.5 | 0.4 | - | 0.8 | - | 0.4 | 0.3 |  | - |
| C_18 : 1_ ω7*c* 11-methyl | 0.1 | 0.3 | - | - | - | - | - |  | - | - | - | - | - |
| C_19 : 0_ cyclo ω8*c* | 3.2 | 3.3 | - | - | - | - | - |  | - | 0.3 | 0.2 |  | 16.3 |
| C_19 : 0_ | 0.5 | - | - | - | - | - | - |  | - | - | - | - | - |
| C_20 : 2_ ω6, 9*c* | 0.1 | - | - | - | - | - | - |  | - | - | -- | - | - |
| Unknown | - | 0.3 | 2.5 | 5.3 | 7.0 | 2.5 | 4.4 | 4.6 | 21.4 | 4.5 | 1.5 | 0.7 | 12.4 |
| Summed feature 2^*^ | 9.6 | 12.2 | 9.0 | 12.2 | 10.0 | 9.8 | 9.8 | 11.0 | - | 8.1 | 8.7 | 7.6 | 10.5 |
| Summed feature 3^*^ | 13.4 | 8.6 | 15.4 | 18.7 | 5.3 | 16.6 | 21.3 | 21.8 | 17.5 | 18.1 | 34.4 | 31.0 | 1.7 |
| Summed feature 5^*^ | 0.6 | - | - | - | - | - | - |  | - | - | 0.6 | - | - |
| Summed feature 8^*^ | 8.6 | 13.1 | 14.3 | 8.5 | 11.3 | 17.3 | 13.6 | 9.5 | 18.8 | 23.6 | 8.6 | 13.6 | 22.8 |
| **Correlation coefficient** | **1.00** | **0.97** | **0.97** | **0.96** | **0.93** | **0.92** | **0.91** | **0.87** | **0.86** | **0.84** | **0.77** | **0.74** | **0.56** |

Strains: **1**, strain PD-1; **2,** *M. intestinalis* DSM 28113^T^; **3,** *M. theicola* QC88-366^T^; **4,** *W toletana* LMG 24162^T^; **5,** *M. calida* LMG 25383^T^; **6**, *M. gaviniae* LMG 25382^T^; **7,** *W. arboricola* BAC 15a-03b^T^; **8,** *M. alhagi* LTYR-11Z^T^; **9,** *W. iniecta* B120^T^; **10**, *P. dispersa* LMG 2603^T^; **11**, *E. amylovora* S59/5^T^; **12**, *P. agglomerans* ATCC 27155^T^; **13,** *P. beijingensis* JZB2120001^T^. Summed feature 2*, 16:1 Iso I/14:0 3OH; Summed feature 3*, 16:1 ω7*c* /16:1 ω6*c*; Summed feature 5*, 18:2 ω6, 9*c*/18:0 ante; Summed feature 8*, 18:1 ω7*c*/18:1 ω7*c*.

*^a^* Data for the type strains taken from ^1^, ^2^, ^3^, ^4^, ^5^, ^6^, and the bacterial diversity (BacDive) database (https://bacdive.dsmz.de/).

**Table S2.** API 20E results for the differentiation of strain PD-1 from closely related strains in the genera *Mixta*, *Erwinia* and *Pantoea*.

| **Tests** | **1** | **2*^a^*** | **3*^a^*** | **4*^a^*** | **5*^a^*** | **6*^a^*** | **7*^a^*** | **8*^a^*** | **9*^a^*** | **10*^a^*** | **11*^a^*** | **12*^a^*** |
| --- | --- | --- | --- | --- | --- | --- | --- | --- | --- | --- | --- | --- |
| Enzyme |  |  |  |  |  |  |  |  |  |  |  |  |
| Arginine dehydrogenase | ± | – | – | – | ± | – | – | – | – | – | ± | – |
| Beta-galactosidase | + | + | + | + | – | + | + | + | + | + | ± | + |
| Gelatinase | – | – | + | – | – | + | + | ± | – | – | + | – |
| Lysine decarboxylase | – | – | – | – | – | – | – | – | – | – | – | – |
| Ornithine decarboxylase | – | – | – | – | + | – | – | – | – | – | – | – |
| Tryptophan deaminase | – | – | – | – | – | – | – | – | – | – | – | – |
| Urease | – | – | – | – | – | – | – | – | – | – | – | – |
| Oxidase | – | – | – | – | – | – | – | – | – | – | – | – |
| Citrate utilization | ± | – | + | – | + | + | + | – | + | – | + | + |
| Acetoin production | – | – | + | + | + | + | + | + | + | + | ± | + |
| H_2_S production | – | – | – | – | – | – | – | – | – | – | – | – |
| Indole production | – | – | – | – | – | – | – | – | – | – | – | + |
| Acid production from (Carbohydrate oxidation) |  |  |  |  |  |  |  |  |  |  |  |  |
| D-Glucose | + | + | + | + | + | + | + | + | + | + | + | + |
| D-Mannitol | + | + | + | + | + | + | + | + | + | + | + | + |
| Inositol | **–** | + | – | + | + | + | + | ± | + | + | + | + |
| Sorbitol | – | – | – | – | – | – | – | ± | + | + | + | + |
| L-Rhamnose | **–** | + | + | + | + | + | + | + | + | + | + | + |
| Sucrose | – | – | + | + | – | + | + | + | + | – | + | + |
| Melibiose | + | + | + | + | + | + | – | ± | + | – | + | + |
| Amygdalin | – | + | – | + | + | + | – | + | + | + | + | + |
| L-Arabinose | + | + | + | + | + | + | + | + | + | + | + | + |
| **Similarity index (SI)*^b^*** | **1** | **0.68** | **0.62** | **0.56** | **0.53** | **0.50** | **0.50** | **0.50** | **0.50** | **0.50** | **0.47** | **0.45** |

Strain names: **1**, strain PD-1; **2**, *M. theicola* QC88-366^T^; **3**, [*Pantoea*] *beijingensis* JZB2120001; **4,** *E. aphidicola* LMG 24877^T^; **5,** *M. tenebrionis* KCTC 72449^T^; **6**, *M. gaviniae* LMG 25382^T^; **7**, *P. vagans* LMG 24199^T^; **8**, *P. agglomerans* DSM 3493^T^, **9**, *E. persicina* LMG 11254^T^, **10**, *E. billingiae* LMG 2613^T^; **11**, *M. calida* LMG 25383^T^; **12**, *P. ananatis* LMG 2665^T^. For strain PD-1 and *M. tenebrionis* KCTC 72449^T^, results from 10 repeated tests performed at 30°C for 24 h are summarized: +, all positive reactions; ±, 10 – 90% positive reactions; -, all negative reactions.

*^a^* Data for the type strains from ^2^ and BacDive database (https://bacdive.dsmz.de/).

*^b^* Similarity index (SI) was calculated by the previous equation (^7^): SI = (*N* – Σ|n_a_-n_b_|)/(*N* + Σ|n_a_-n_b_|), where *N* is the number of paired API 20E test sets (*N* = 21) and the term Σ|n_a_-n_b_| is the sum of absolute difference in each test between strains. ND, not determined.

**Table S3.** API 50CHB/E test results for the comparison between strain PD-1 and the closest strain [*Pantoea*] *beijingensis* JZB2120001.

| **Characteristics** | **Strain PD-1** | | **[*Pantoea*]**  ***beijingensis*** *^a^* | **Characteristics** | **Strain PD-1** | | **[*Pantoea*]**  ***beijingensis*** *^a^* |
| --- | --- | --- | --- | --- | --- | --- | --- |
|  | **24 h** | **48 h** |  |  | **24 h** | **48 h** |  |
| 50CHB/E medium control | – | – |  | Esculin ferric citrate | – | – | + |
| Glycerol | – | + | – | Salicin | – | – | + |
| Erythritol | – | + | – | D-Cellobiose | – | – | – |
| D-Arabinose | V | + | – | D-Maltose | + | + | + |
| L-Arabinose | V | + | + | D-Lactose | – | – | – |
| D-Ribose | + | + | + | D-Melibiose | + | + | + |
| D-Xylose | + | + | + | Sucrose | – | – | + |
| L-Xylose | – | – | – | D-Trehalose | + | + | + |
| D-Adonitol | – | – | – | Inulin | – | – | – |
| Methyl-β-D-xylopyranoside | – | – | – | D-Melezitose | – | – | – |
| D-Galactose | + | + | + | D-Raffinose | – | – | – |
| D-Glucose | + | + | + | Amidon (starch) | – | – | – |
| D-Fructose | + | + | + | Glycogen | – | – | – |
| D-Mannose | + | + | + | Xylitol | – | – | – |
| L-Sorbose | – | – | – | Gentiobiose | + | V | + |
| L-Rhamnose | – | – | + | D-Turanose | – | – | – |
| Ducitol | – | – | – | D-Lyxose | – | – | + |
| Inositol | – | – | – | D-Tagatose | – | – | – |
| D-Mannitol | + | + | + | D-Fucose | V | + | – |
| D-Sorbitol | – | – | – | L-Fucose | – | + | – |
| Methyl-α-D-mannopyranoside | – | – | – | D-Arabitol | + | + | – |
| Methyl-α-D-glucopyranoside | – | – | – | L-Arabitol | – | – | – |
| N-acetyl-glucosamine | + | + | + | Gluconate, K | – | – | – |
| Amygdalin | – | – | – | 2-Ketogluconate, K | – | – | – |
| Arbutin | – | – | + | 5-Ketogluconate, K | – | – | – |

*^a^* Data taken from ^2^. For the strain PD-1, API 50CHB/E test results were recorded at 30°C for 24 h and 48 h according to the API manual.

**Table S4.** Glycosyl hydrolase (GH) family proteins encoded in the genome of strain PD-1

| **Gene ID**  **(locus tag)** | **GH family** | **Homolg ID**  **(NCBI)** | **Identity (%)** | **E-values** | **Enzymes in Family** |
| --- | --- | --- | --- | --- | --- |
| PMPD1_2478 | GH24 | AWQ18457.1 | 73.3 | 1.3E-94 | lysozyme (EC 3.2.1.17) |
| PMPD1_2404 | GH103 | ARJ43469.1 | 75.1 | 0 | peptidoglycan lytic transglycosylase  (EC 3.2.1.-) |
| PMPD1_1986 | GH94 | ASV54592.1 | 83.5 | 0 | cellobiose phosphorylase (EC 2.4.1.20) |
| PMPD1_1970 | GH88 | AKE58954.1 | 69.2 | 0 | d-4,5-unsaturated β-glucuronyl hydrolase (EC 3.2.1.-) |
| PMPD1_1883 | GH37 | ARJ43888.1 | 83.2 | 0 | α,α-trehalase (EC 3.2.1.28). |
| PMPD1_1838 | GH3 | ADU68814.1 | 82.1 | 0 | β-glucosidase (EC 3.2.1.21) |
| PMPD1_1814 | GH73 | ADU68788.1 | 74.1 | 2.06E-174 | lysozyme (EC 3.2.1.17) |
| PMPD1_1529 | GH3 | CAX58907.1 | 86.7 | 0 | β-glucosidase (EC 3.2.1.21) |
| PMPD1_1516 | GH15 | QBR51047.1 | 79.0 | 0 | glucoamylase (EC 3.2.1.3) |
| PMPD1_1482 | GH13_29 | QAR47257.1 | 76.0 | 0 | α-amylase (EC 3.2.1.1) |
| PMPD1_1429 | GH20 | AXF75237.1 | 72.7 | 0 | β-hexosaminidase (EC 3.2.1.52) |
| PMPD1_1327 | GH109 | QDK77782.1 | 24.2 | 9.66E-13 | α-N-acetylgalactosaminidase (EC 3.2.1.49) |
| PMPD1_1148 | GH2 | AMG57672.1 | 70.0 | 0 | β-galactosidase (EC 3.2.1.23) |
| PMPD1_1135 | GH1 | AMB74883.1 | 76.2 | 0 | β-glucosidase (EC 3.2.1.21) |
| PMPD1_1097 | GH109 | SDU84370.1 | 31.0 | 5.06E-11 | α-N-acetylgalactosaminidase (EC 3.2.1.49) |
| PMPD1_0983 | GH23 | CUU23073.1 | 82.6 | 0 | lysozyme type G (EC 3.2.1.17) |
| PMPD1_0729 | GH73 | AVV37563.1 | 64.1 | 0 | lysozyme (EC 3.2.1.17) |
| PMPD1_0543 | GH20 | ADU67852.1 | 70.0 | 0 | β-hexosaminidase (EC 3.2.1.52) |
| PMPD1_0542 | GH13_29 | AUX92143.1 | 88.2 | 0 | α-amylase (EC 3.2.1.1) |
| PMPD1_0540 | GH4 | QBR49249.1 | 84.8 | 0 | maltose-6-phosphate glucosidase (EC 3.2.1.122) |
| PMPD1_0369 | GH13_36 | AEA21393.1 | 54.1 | 0 | α-amylase (EC 3.2.1.1) |
| PMPD1_0304 | GH77 | AUY23761.1 | 76.6 | 0 | amylomaltase or 4-α-glucanotransferase (EC 2.4.1.25) |
| PMPD1_0294 | GH13_11 | AIX75593.1 | 76.7 | 0 | α-amylase (EC 3.2.1.1) |
| PMPD1_0293 | GH13_9 | AUX91928.1 | 86.7 | 0 | α-amylase (EC 3.2.1.1) |
| PMPD1_0145 | GH8 | CAB89803.1 | 70.6 | 3.87E-174 | chitosanase (EC 3.2.1.132) |
| PMPD1_3691 | GH109 | SDU84370.1 | 39.1 | 8.15E-18 | α-N-acetylgalactosaminidase (EC 3.2.1.49) |
| PMPD1_3619 | GH1 | QBR48966.1 | 90.4 | 0 | β-glucosidase (EC 3.2.1.21) |
| PMPD1_3562 | GH102 | AIX72703.1 | 87.2 | 0 | peptidoglycan lytic transglycosylase (EC 3.2.1.-) |
| PMPD1_3443 | GH103 | AXU93907.1 | 74.7 | 0 | peptidoglycan lytic transglycosylase (EC 3.2.1.-) |
| PMPD1_3327 | GH23 | CUU24932.1 | 85.6 | 0 | lysozyme type G (EC 3.2.1.17) |
| PMPD1_3099 | GH24 | AWP32170.1 | 77.8 | 3.27E-93 | lysozyme (EC 3.2.1.17) |
| PMPD1_3079 | GH23 | AVX39032.1 | 71.9 | 0 | lysozyme type G (EC 3.2.1.17) |
| PMPD1_3076 | GH33 | AGF89170.1 | 26.0 | 1.07E-27 | sialidase or neuraminidase (EC 3.2.1.18) |
| PMPD1_2625 | GH1 | AMO82759.1 | 79.5 | 0 | β-glucosidase (EC 3.2.1.21) |
| PMPD1_2619 | GH13_5 | CUU24442.1 | 80.1 | 0 | α-amylase (EC 3.2.1.1) |
| PMPD1_2586 | GH88 | AKE58954.1 | 75.1 | 0 | d-4,5-unsaturated β-glucuronyl hydrolase (EC 3.2.1.-) |

**Figure S1.** UPGMA phylogenetic tree of 16S rRNA genes of strain PD-1 and type strains of closely related species. The optimal tree is shown with more than 75% of 1000 replicate trees in the bootstrap test. The scale shows the branch lengths as the evolutionary distances computed by the Maximum Composite Likelihood method in MEGA X and the dotted lines (a and b) indicate the similarity threshold of 98.7% for species and 95% for genus (^8^). The numbers in parentheses represent the accession numbers of 71 16S rRNA gene sequences obtained from Silva rRNA database (https://www.arb-silva.de/) and 2 genomes (strains WGM and LNK).

**Figure S2.** UPGMA phylogenetic tree of *Erwiniaceae* species based on concatenated nucleotide sequences of *atpD*, *gyrB*, *infB* and *rpoB* genes annotated on the reference genomes in Supplementary Data. *Enterobacter cloacae* ATCC 13047^T^ was the outgroup species. The optimal tree is shown with >90% values of 1000 replicate trees in the bootstrap test. The scale shows the unit of the numbers of nucleotide substation per site as the evolutionary distance computed using the Maximum Composite Likelihood method in MEGA X. This analysis included a total of 10,478 positions in the final dataset of 40 nucleotide sequences with the lengths of concatenated coding DNA regions (bp) in parentheses. Codon positions included were 1st+2nd+3rd+Noncoding. All positions with less than 95% site coverage were eliminated, i.e., fewer than 5% alignment gaps, missing (incomplete or frameshifted) data, and ambiguous bases were allowed at any position (partial deletion option).


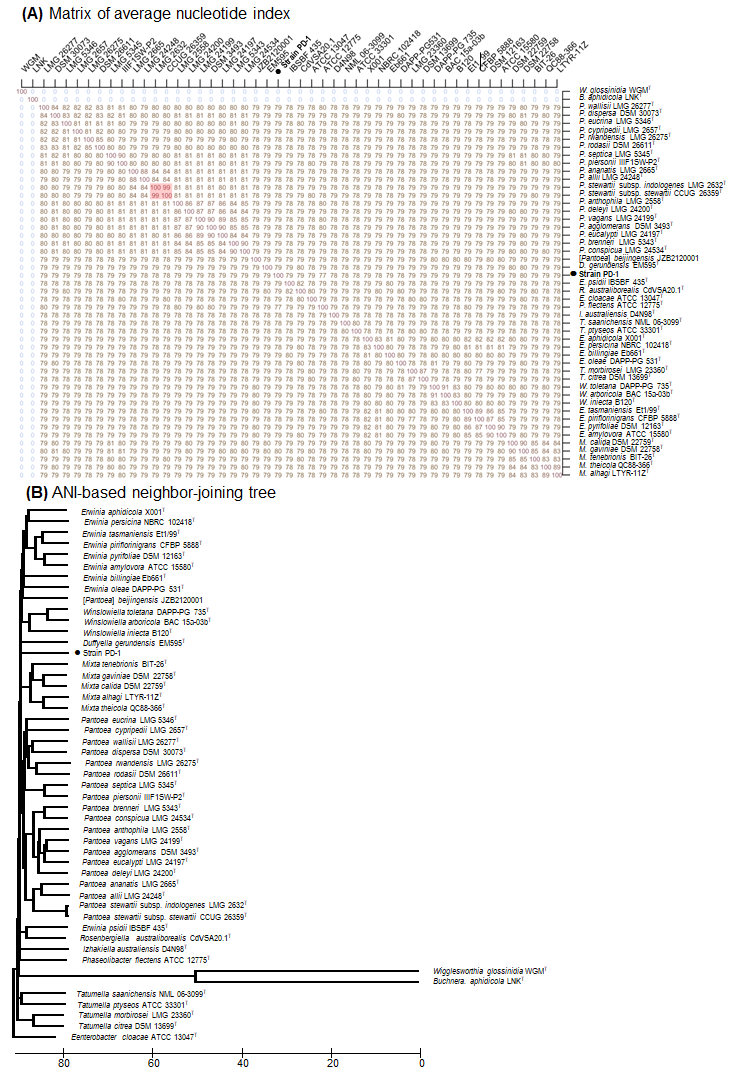


Figure S3 Genomic analysis of strain PD-1 and type strains of *Erwiniaceae* species.

(A) Average nucleotide index (ANI) matrix constructed through the pairwise comparison of genomes in the Supplementary Data. (B) A Neighbor-joining tree constructed with ANI-derived distance measures between genomes. The scale was determined as the evolutionary distances of genomes. *Enterobacter cloacae* ATCC 13047^T^ was the outgroup species. ANI values below 75% (zero values in the reciprocal crosses) are not to be trusted, and AAI should be used instead in Figure S4 according to an introductory comment from the Kostas Lab (^9^).


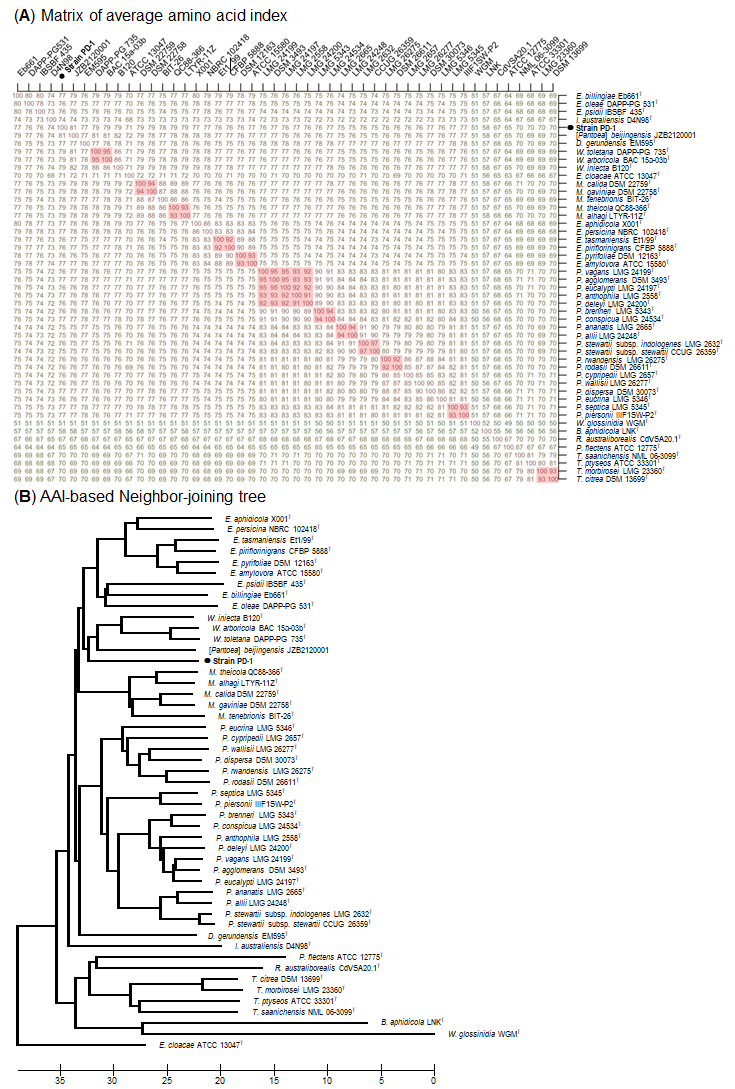


Figure S4. Analysis of average amino acid (AAI) index between strain PD-1 and type strains of *Erwiniaceae* species.

(A) AAI matrix constructed through the pairwise comparison of protein sequence sets obtained from the translation of genomes in the Supplementary Data. Gene annotation was performed by the NCBI Prokaryotic Genome Annotation Pipeline (^10^). (B) A Neighbor-joining tree constructed with AAI-derived distance measures between strains. The scale was determined as the evolutionary distances of amino acid sequences calculated by BLAST-based average amino acid identity. *Enterobacter cloacae* ATCC 13047^T^ was the outgroup species.

**Figure S5. Detection, purification, and quantification of the major respiratory quinones from *Enterobacteriaceae* and *Erwiniaceae* strains.** Ubiquinone 10 (Q10) was included as an exogeneous standard in sample analysis. (A) Peaks of three respiratory quinones (Q8, DMK8, and MK8) and a carotenoid (zeaxanthin [M-H]^+^ at *m/z* 570), extracted from aerobically and anaerobically grown cells of strains described in the Methods part, were identified by HPLC with UV detection at 254 nm and mass spectrometry. (B) Standard curves of three major quinones (Q8, DMK8, and MK8) of *Escherichia coli* K-12 MG1655. Each quinone compound was purified by using a preparative ZORBAX C18 and repeated chromatography with UV detection at 254 nm for the standard curve, as shown at the right plot.


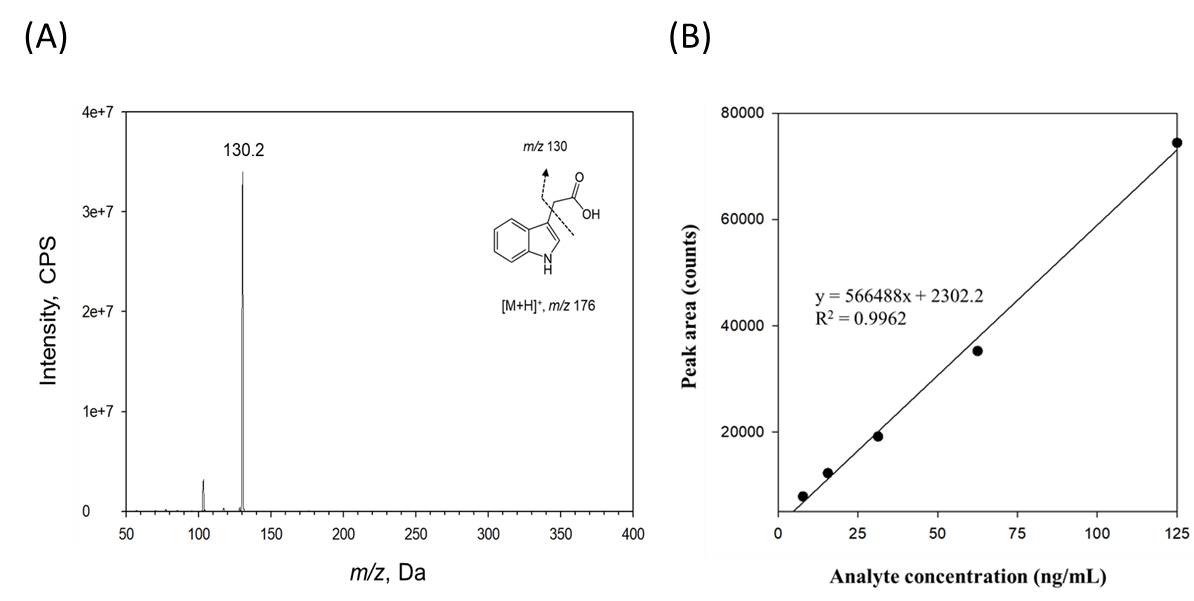


**Figure S6.** LC-MS/MS spectrum (A) and standard curve (B) for indole-3-acetic acid (IAA). MRM: 176 🡪 130

**Supplementary References**

1 Feistner, G. J. & Ishimaru, C. Proferrioxamine profiles of Erwinia herbicola and related bacteria. *BioMetals* **9**, 337-344 (1996).

2 Liu, Y. *et al.* Pantoea beijingensis sp. nov., isolated from the fruiting body of Pleurotus eryngii. *Antonie Van Leeuwenhoek* **104**, 1039-1047 (2013).

3 Campillo, T. *et al.* Erwinia iniecta sp. nov., isolated from Russian wheat aphid (Diuraphis noxia). *International Journal of Systematic and Evolutionary Microbiology* **65**, 3625-3633 (2015).

4 Prakash, O. *et al.* Pantoea intestinalis sp. nov., isolated from the human gut. *International journal of systematic and evolutionary microbiology* **65**, 3352-3358 (2015).

5 Chen, C. *et al.* Pantoea alhagi, a novel endophytic bacterium with ability to improve growth and drought tolerance in wheat. *Scientific Reports* **7**, 41564 (2017).

6 Brady, C., Kaur, S., Crampton, B. & Arnold, D. Transfer of Erwinia toletana and Erwinia iniecta to a novel genus Winslowiella gen. nov. as Winslowiella toletana comb. nov. and Winslowiella iniecta comb. nov. and description of Winslowiella arboricola sp. nov., isolated from bleeding cankers on broadleaf hosts. *Frontiers in Microbiology* **13**, 1063107 (2022).

7 Pham, H. *et al.* Analysis of phylogenetic markers for classification of a hydrogen peroxide producing Streptococcus oralis isolated from saliva by a newly devised differential medium. *Journal of Microbiology* **60**, 795-805 (2022).

8 Rossi-Tamisier, M., Benamar, S., Raoult, D. & Fournier, P.-E. Cautionary tale of using 16S rRNA gene sequence similarity values in identification of human-associated bacterial species. *International journal of systematic and evolutionary microbiology* **65**, 1929-1934 (2015).

9 Rodriguez-R, L. M. & Konstantinidis, K. T. The enveomics collection: a toolbox for specialized analyses of microbial genomes and metagenomes. Report No. 2167-9843, (PeerJ Preprints, 2016).

10 Tatusova, T. *et al.* NCBI prokaryotic genome annotation pipeline. *Nucleic acids research* **44**, 6614-6624 (2016).
